# PGRMC1 acts as a size-selective cargo receptor to drive ER-phagic clearance of mutant prohormones

**DOI:** 10.1038/s41467-021-26225-8

**Published:** 2021-10-13

**Authors:** Yu-Jie Chen, Jeffrey Knupp, Anoop Arunagiri, Leena Haataja, Peter Arvan, Billy Tsai

**Affiliations:** 1 Department of Cell & Developmental Biology, University of Michigan Medical School, 109 Zina Pitcher Place, BSRB 3043, Ann Arbor, MI 48109 USA; 2 Cellular and Molecular Biology Program, University of Michigan Medical School, Ann Arbor, MI USA; 3 Division of Metabolism Endocrinology & Diabetes, University of Michigan Medical School, Ann Arbor, MI USA

**Keywords:** Protein folding, Autophagy, Endoplasmic reticulum, Diabetes

## Abstract

The reticulon-3 (RTN3)-driven targeting complex promotes clearance of misfolded prohormones from the endoplasmic reticulum (ER) for lysosomal destruction by ER-phagy. Because RTN3 resides in the cytosolic leaflet of the ER bilayer, the mechanism of selecting misfolded prohormones as ER-phagy cargo on the luminal side of the ER membrane remains unknown. Here we identify the ER transmembrane protein PGRMC1 as an RTN3-binding partner. Via its luminal domain, PGRMC1 captures misfolded prohormones, targeting them for RTN3-dependent ER-phagy. PGRMC1 selects cargos that are smaller than the large size of other reported ER-phagy substrates. Cargos for PGRMC1 include mutant proinsulins that block secretion of wildtype proinsulin through dominant-negative interactions within the ER, causing insulin-deficiency. Chemical perturbation of PGRMC1 partially restores WT insulin storage by preventing ER-phagic degradation of WT and mutant proinsulin. Thus, PGRMC1 acts as a size-selective cargo receptor during RTN3-dependent ER-phagy, and is a potential therapeutic target for diabetes.

## Abstract (web summary)

Degradation of misfolded proteins from the endoplasmic reticulum (ER) is important for maintaining proper cellular protein homeostasis. Here, the authors discovered that the ER membrane protein PGRMC1 binds to misfolded prohormones for recruitment into the ER-phagy degradative pathway.

## Introduction

Protein misfolding in the endoplasmic reticulum (ER) causes a myriad of human diseases, including neurodegenerative diseases, cancer, and diabetes^1–4^. To remove proteins with toxic misfolding, cells harbor an intricate network of protein quality control pathways designed to target misfolded proteins for proteasomal or lysosomal destruction^5–9^. As approximately one-third of the entire cellular proteome is translocated into the ER, it is not surprising that this compartment is endowed with elaborate protein quality control machineries designed for misfolded protein clearance.

Amongst the most well characterized of the ER quality control pathways is ER-associated protein degradation (ERAD). During ERAD, select ER membrane protein complexes (including the well-characterized Hrd1-Derlin complex) conduct misfolded luminal or membrane cargos to the cytosol where they are proteasomally degraded^10–13^. However, whereas ERAD typically removes soluble cargos via retrotranslocation of one protein at a time^14^, ER protein aggregates are targeted for lysosomal destruction via ER-coupled autophagy (ER-phagy)^15,16^.

In ER-phagy, an ER membrane targeting complex lies at the crucial ER-cytosol interface. The targeting complex is strategically positioned to allow for accumulation of luminally selected cargo, and this organelle subregion is presented to the cytosolic autophagy machinery for degradation^15,16^. To date, six ER membrane proteins (FAM134B, RTN3, ATL3, CCPG1, SEC62, and TEX264) have been shown to participate in distinct ER-phagy targeting complexes in mammals^15,17–24^. Importantly, all of them harbor the cytosolic LC3-interacting region (LIR) that is used to engage LC3 (which is anchored within the phagophore membrane) to initiate the ER-phagic process. By contrast, how these targeting complexes participate in the capture of cargo localized within the ER lumen has been largely unclear. In one instance, the calnexin transmembrane protein was found to link ER cargo to the FAM134B ER-phagy targeting complex^21^, but the mechanistic basis by which other targeting complexes identify their respective cargos remains entirely unknown.

We previously found that RTN3 participates in an ER-phagy targeting complex to promote lysosomal clearance of two misfolded prohormones: mutant proopiomelanocortin [POMC, which is responsible for hyperphagia and severe obesity^25^], and mutant proinsulin [which causes a debilitating diabetic syndrome called mutant *INS*-gene-induced diabetes of youth (MIDY)^26–29^]. Specifically, loss-of-function studies revealed that depletion of RTN3 (but not other ER-phagy targeting complexes including FAM134B, which is structurally and topologically similar to RTN3) blocked the degradation of mutant POMC and proinsulin^30^, indicating that RTN3 is responsible for the clearance of a large portion of these misfolded prohormones. However, because RTN3 is inserted into the cytosolic leaflet of the ER membrane^31,32^, the targeting complex still requires additional machinery to capture luminally localized cargos. We therefore hypothesized that an RTN3-interacting ER membrane component functions to recruit the misfolded prohormones for ER-phagy. Given the established role of ER-phagy in the disposal of collagens that are misfolded into massive fibrils^21^, one might assume that the misfolded prohormone cargo delivered to ER-phagy would also be comprised of massive, megadalton protein aggregates.

Here, using an unbiased proteomics approach, we have identified the ER transmembrane protein Progesterone Receptor Membrane Component 1 (PGRMC1) as an RTN3-interacting partner. We show that, via its luminal domain, PGRMC1 associates with mutant prohormones and delivers them for RTN3-dependent ER-phagic clearance. Remarkably, we find that the PGRMC1 pathway involves soluble cargos whose core components are smaller (< 150 kDa) in size than ER-phagy substrates that have previously been reported. In the case of a MIDY mutant proinsulin that entraps WT proinsulin in the ER resulting in defective insulin storage, we find that chemical inactivation of PGRMC1 partially restores WT insulin storage, accompanied by enhanced insulin secretion. Hence, PGRMC1 acts as a size-selective cargo receptor for RTN3-dependent ER-phagy, and represents a potential therapeutic target for diseases involving peptide prohormone misfolding.

## Results

### The ER-phagy targeting complex involves RTN3 engagement of PGRMC1

**Fig. 1:**
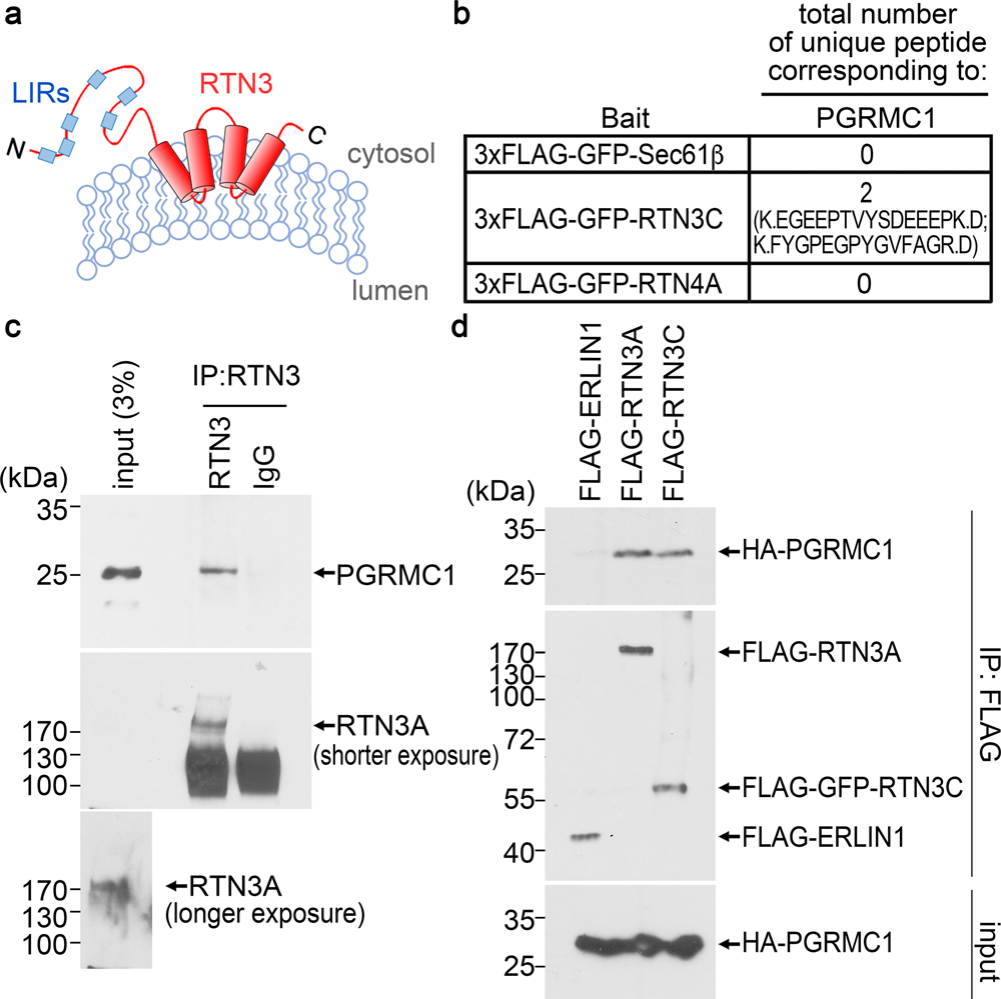
The ER-phagy targeting complex involves RTN3 engagement of PGRMC1. **a** Schematic of the topology of RTN3. **b** Total number of peptides corresponding to PGRMC1 identified by mass spectrometry on materials obtained from FLAG IP of HEK 293 T cells expressing 3xFLAG-GFP-Sec61B, 3xFLAG-GFP-RTN3C, or 3xFLAG-GFP-RTN4A. **c** HEK 293 T cells were treated with the cross-linker DSP and subject to lysis. IP was performed using either anti-RTN3 or IgG antibody, and the precipitated material was subjected to SDS-PAGE followed by immunoblotting with the indicated antibodies. *N* = 3 independent experiments. **d** HEK 293 T cells expressing HA-PGRMC1 were co-transfected with FLAG-ERLIN1, FLAG-RTN3A, or FLAG-RTN3C. Cells were lysed and the resulting lysate incubated with a FLAG antibody. The immunoprecipitated material was eluted with excess 3xFLAG, subjected to SDS-PAGE followed by immunoblotting with the indicated antibodies. *N* = 3 independent experiments. Source data are provided as a Source Data file.

The RTN3 targeting complex delivers misfolded mutant prohomones for ER-phagic lysosomal degradation^30^, yet RTN3 itself cannot bind directly to luminally localized ER cargos because RTN3 is topologically positioned within the cytosolic leaflet of the ER membrane (Fig. 1a). We therefore postulated that RTN3 must directly or indirectly interact with a bona fide ER transmembrane receptor to promote ER-phagic clearance of selected mutant prohomones. To facilitate biochemical isolation of RTN3-binding partner(s), a 3xFLAG-GFP cassette was appended to the N-terminus of RTN3C, generating 3xFLAG-GFP-RTN3C, which is known to be functional^30^. 3xFLAG-GFP-Sec61β was used as a negative control because Sec61β is an ER transmembrane protein with no reported role in ER-phagy. Furthermore, because RTN4 adopts a similar topology as RTN3 but does not mediate ER-phagic clearance of mutant prohormones^30^, 3xFLAG-GFP-RTN4A serves as an additional control; RTN4A refers to full-length RTN4 harboring the cytosolic domain.

3xFLAG-GFP-RTN3C, 3xFLAG-GFP-Sec61β, or 3xFLAG-GFP-RTN4A transiently expressed individually in HEK293T cells were each immunoprecipitated with anti-FLAG antibody and the immunoprecipitate specifically eluted with excess 3x FLAG peptide. Mass spectrometry (MS) analysis of the eluates revealed two peptides matching PGRMC1 released specifically from 3xFLAG-GFP-RTN3C (Fig. 1b), suggesting that PGRMC1 complexes with RTN3. (The entire mass spectrometry data is available in Table S1). Moreover, we found that endogenous full-length RTN3 (RTN3A) co-precipitated endogenous PGRMC1 under crosslinking conditions (Fig. 1c). The PGRMC1 interaction is not mediated by the RTN3A cytosolic N-terminal domain (NTD) because immunoprecipitation (IP) of FLAG-RTN3C, lacking the RTN3-NTD, also co-IP’d HA-PGRMC1 at comparable levels (Fig. 1d), whereas IP of the control ER membrane protein FLAG-ERLIN1 did not pull down HA-PGRMC1 (Fig. 1d). These findings establish that PGRMC1 is recruited to the RTN3 targeting complex.

### PGRMC1 binds to mutant POMC and promotes its RTN3-dependent ER-phagic clearance

**Fig. 2:**
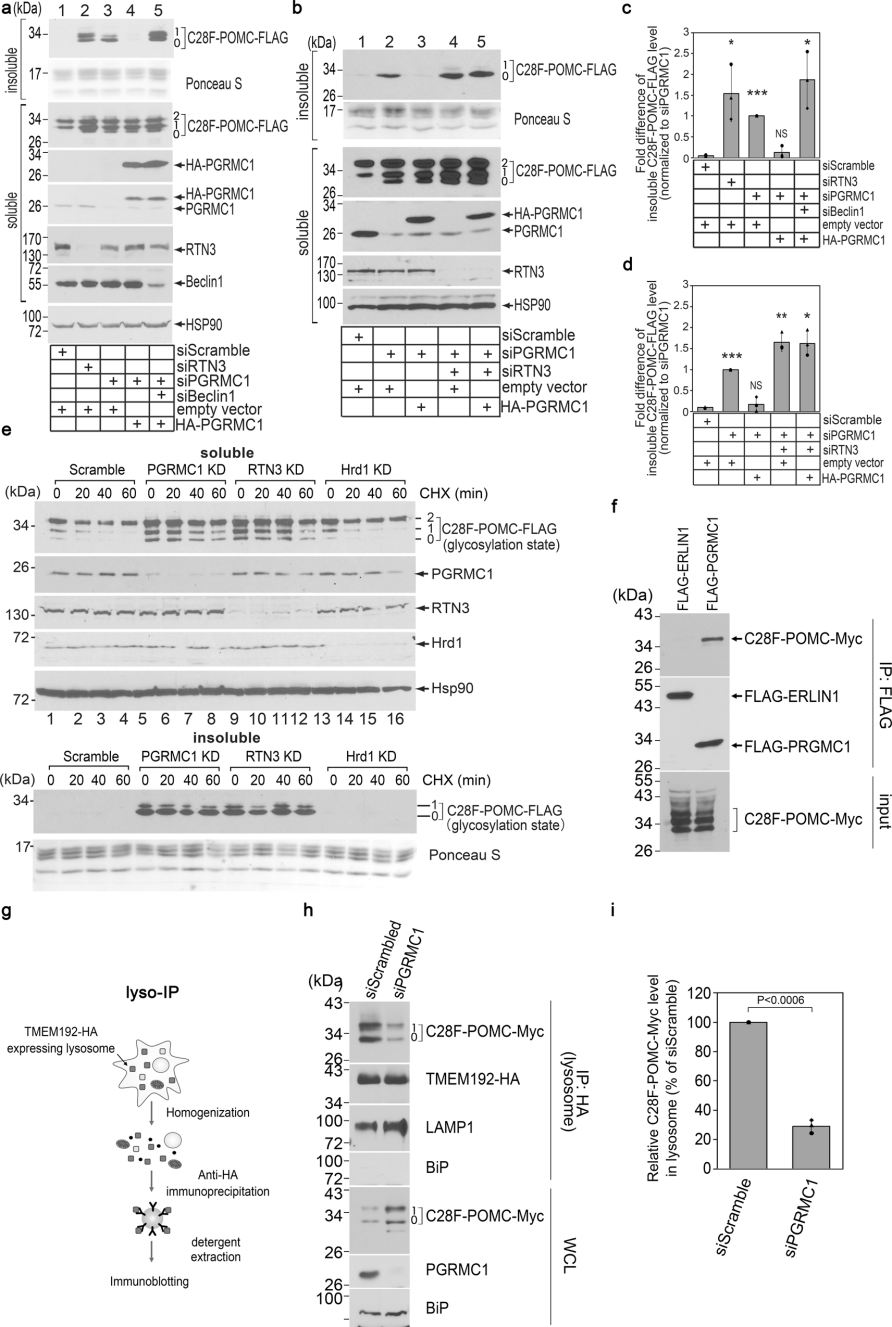
PGRMC1 binds to mutant POMC and promotes its RTN3-dependent ER-phagic clearance. **a** HEK 293 T cells expressing C28F-POMC-FLAG and either empty vector or HA-PGRMC1 were transfected with the indicated siRNAs. Cells were solubilized with RIPA buffer (containing the conventional detergents 1% Triton X-100, 0.5% sodium deoxycholate, and 0.1% SDS) to generate a “soluble” fraction. The insoluble material was subsequently extracted by 2% SDS and is referred to as the “insoluble” fraction. Both the soluble and insoluble fractions were subjected to SDS-PAGE and immunoblotted as indicated. *N* = 3 independent experiments. **b** As in **a** but using the indicated siRNAs. *N* = 3 independent experiments. **c** Quantification of the insoluble C28F-POMC-FLAG level from **a**, with the protein level relative to the PGRMC1 siRNA-treated condition (lane 2). Data are represented as mean ± SD. *N* = 3 independent experiments. One-tailed Standard Student’s *t*-test was used to determine statistical significance. From left to right, corresponding p-values are: < 0.028, < 0.0001, < 0.176, < 0.02. **d** Quantification of the insoluble C28F-POMC-FLAG level from **b**, with the protein level relative to the PGRMC1 siRNA condition (lane 3). Data are represented as mean ± SD. *N* = 3 independent experiments. One-tailed Standard Student’s *t*-test was used to determine statistical significance. From left to right, corresponding *p*-values are: < 0.0001, < 0.272, < 0.003, < 0.006. **e** HEK 293 T cells expressing C28F-POMC-FLAG and transfected with the indicated siRNAs were treated with cycloheximide for 0, 20, 40, 60 min. The soluble (top) and insoluble (bottom) materials were collected as in **a** and subject to SDS-PAGE and immunoblotted with the indicated antibodies. *N* = 3 independent experiments. **f** HEK 293 T cells were transfected with C28F-POMC-Myc and either FLAG-ERLIN1 or FLAG-PGRMC1. Cells were lysed and immunoprecipitated using a FLAG antibody. Whole-cell lysate (input) and 3xFLAG peptide eluate was subject to SDS-PAGE and immunoblotted as indicated. *N* = 3 independent experiments. **g** A schematic of the lyso-IP protocol, as described in method^34^. **h** Lyso-IP was performed on HEK 293 T cells transfected with C28F-POMC-Myc and co-transfected with either scramble or PGRMC1 siRNA. Samples were subjected to SDS-PAGE and immunoblotted as indicated. *N* = 3 independent experiments. **i** Quantification of C28F-POMC-FLAG in the immunoprecipitated material from **h** (top panel). *N* = 3 independent experiments. One-tailed Standard Student’s *t*-test was used to determine statistical significance. *P ≤ 0.05; **P ≤ 0.005; ***P ≤ 0.001. Data are represented as mean ± SD. Source data are provided as a Source Data file. See also Fig. S1.

Because RTN3 triggers ER-phagic clearance of the mutant prohormone C28F-POMC^30^, our identification of PGRMC1 as an RTN3 partner prompted us to ask whether PGRMC1 also promotes degradation of this misfolded prohormone. We previously established a biochemical assay to probe the fate of C28F-POMC-FLAG in control and (siRNA-mediated) RTN3 knockdown (KD) HEK293T cells^30^. In this assay, C28F-POMC-FLAG-expressing cells were subjected to detergent extraction generating a “soluble” fraction extracted by RIPA buffer (containing 1% Triton X-100, 0.5% sodium deoxycholate, and 0.1% SDS) and an “insoluble” fraction that was resistant to RIPA extraction but extracted in a harsher detergent (2% SDS). Using this approach, we found that the level of undegraded C28F-POMC-FLAG increased robustly in insoluble (and soluble) fractions of RTN3 KD cells (Fig. 2a, lane 2 vs 1; quantified in Fig. 2c), confirming that RTN3 promotes clearance of this mutant prohormone^30^.

Importantly, the level of C28F-POMC-FLAG also increased markedly under PGRMC1 KD (Fig. 2a lane 3 vs 1; quantified in Fig. 2c), indicating that PGRMC1 is also required for clearance of this mutant prohormone cargo. By contrast, loss of TMEM97 did not result in C28F-POMC accumulation (Fig. S1a), indicating a function for PGRMC1 that is independent of this reported binding partner^33^. Under PGRMC1 KD conditions, re-expression of (siRNA-resistant) HA-PGRMC1 construct restored clearance C28F-POMC-FLAG (Fig. 2a lane 4 vs 3; quantified in Fig. 2c). However, additional depletion of the major autophagy component Beclin1 in PGRMC1 KD cells precluded HA-PGRMC1 from facilitating the degradation of C28F-POMC-FLAG (Fig. 2a lane 5 vs 4; quantified in Fig. 2c). Further, whereas expression of HA-PGRMC1 in PGRMC1 KD cells enabled clearance of C28F-POMC-FLAG (Fig. 2b lane 3 vs 2; quantified in Fig. 2d), additional depletion of RTN3 in the PGRMC1 KD cells prevented HA-PGRMC1 from facilitating the degradation of C28F-POMC-FLAG (Fig. 2b lane 5 vs 4; quantified in Fig. 2d). These results indicate that the PGRMC1 clearance pathway of the C28F-POMC-FLAG cargo is autophagy-dependent, utilizing RTN3-dependent ER-phagy.

We used translational shutoff [cycloheximide (CHX) chase] to further test that PGRMC1 promotes C28F-POMC-FLAG turnover. At the beginning of the experiment (T_0_), the level of soluble C28F-POMC-FLAG was already increased by knockdown of PGRMC1 or RTN3 (but not Hrd1; Fig. 2e top, lanes 5, 9 vs 1, 13). Particularly affected were nonglycosylated (“0”) and singly glycosylated (1”) forms of the substrate (quantified in Fig. S1b). [When C28F-POMC-FLAG expressed in control cells was treated with endoH (which removes only ER-modified N-linked glycans) or PGNase F (which removes all N-linked glycans), both singly glycosylated and doubly-glycosylated (“2”) forms were completely digested (Fig. S1c), indicating that both are retained in the ER.] During the CHX chase, degradation of C28F-POMC-FLAG was impaired upon PGRMC1 or RTN3 depletion (Fig. 2e) in the soluble fraction (quantified in Fig. S1d) and the insoluble fraction (quantified in Fig. S1e). These findings are consistent with the idea that PGRMC1, a transmembrane protein spanning the ER bilayer, executes a critical function in targeting C28F-POMC as cargo for ER-phagic degradation. Indeed, we found that IP of PGRMC1 with a FLAG-tagged cytosolic NTD (FLAG-PGRMC1) but not a control ER membrane protein (FLAG-ERLIN1) could co-IP C28F-POMC-Myc (Fig. 2f). These data suggest that the mutant prohormone residing within the ER lumen is recruited selectively by PGRMC1 to initiate ER-phagy (established further below).

To test whether delivery of C28F-POMC to lysosomes depends on PGRMC1, we used an established lysosome-IP (lyso-IP) approach to immunoisolate lysosomes^34^ (Fig. 2g). In transiently transfected cells that were mechanically homogenized (detergent-free), organelles immunoisolated from the whole-cell lysate (WCL) were analyzed by SDS-PAGE and immunoblotting. The immunoisolated organelles contained the transiently expressed lysosomal marker TMEM192-HA (and another lysosomal marker, LAMP1), but were devoid of the ER protein BiP (Fig. 2h), indicating enrichment of lysosomes without significant ER contamination. Importantly, whereas PGRMC1 KD robustly increased the level of C28F-POMC-Myc in the WCL, C28F-POMC-Myc delivery to lysosomes was dramatically decreased (Fig. 2h; quantified in 2i). We further probed the role of PGRMC1 in cargo delivery to the lysosome by confocal microscopy and found that under control conditions, C28F-POMC-Myc exhibits substantial colocalization with TMEM192-HA, indicating flux of mutant POMC to lysosomes (Fig. S1f, top panels). However, when PGRMC1 is knocked down, large C28F-POMC puncta form, with diminished colocalization with TMEM192-HA (Fig. S1f, bottom panels). Together, these findings demonstrate that PGRMC1 is required to direct the ER-localized mutant prohormone for degradation via an RTN3-dependent ER-phagy targeting complex that delivers cargo to lysosomes.

If PGRMC1 promotes clearance of ER-retained C28F-POMC, it is possible that loss of PGRMC1 may allow ER exit and anterograde trafficking of C28F-POMC. However, we found that under PGRMC1 knockdown, C28F-POMC-FLAG is still completely sensitive to endoH treatment (Fig. S1g, compare lane 2 to 1). Therefore, depletion of PGRMC1 blocks the turnover of C28F-POMC but does not override quality control of anterograde ER export for the misfolded mutant POMC.

### PGRMC1 uses its luminal domain to recruit cargo

**Fig. 3:**
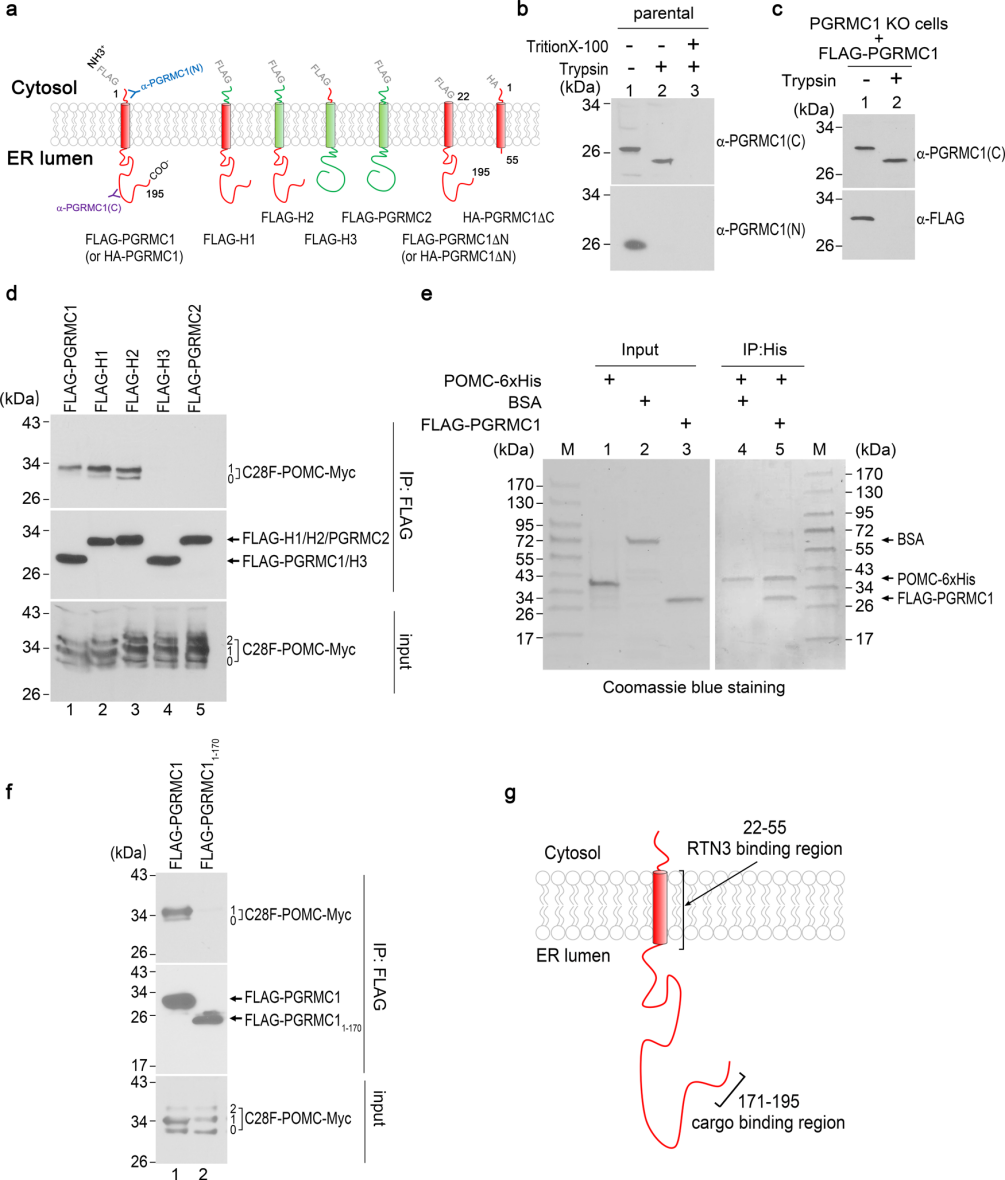
PGRMC1 uses its luminal domain to recruit cargo. **a** Schematic of the topology of PGRMC1, PGRMC2, and the hybrid and mutant variants generated. **b** HEK 293 T cells were mechanically disrupted and the membranes were pelleted. Membranes treated with or without trypsin (and Triton-X-100) were subject to SDS-PAGE and immunoblotted as indicated. *N* = 3 independent experiments. **c** As in **b**, except HEK293T PGRMC1 knockout (KO) cells transfected with FLAG-PGRMC1 were used. *N* = 3 independent experiments. **d** HEK293T cells expressing C28F-POMC-Myc and either FLAG-PGRMC1, FLAG-Hybrid 1 (H1), FLAG-Hybrid 2 (H2), FLAG-Hybrid 3 (H3), or FLAG-PGRMC2 were lysed and FLAG IP was performed as in Fig. 2f, followed by SDS-PAGE and immunoblotting. *N* = 3 independent experiments. **e** *E. coli* purified nonglycosylated POMC-6xHis (Abcam) was incubated with either FLAG-PGRMC1 (purified from HEK 293 T cells) or BSA as a control. Samples were precipitated using magnetic NTA beads, washed extensively, and analyzed by SDS-PAGE with subsequent Coomassie blue staining. ‘M’ indicates protein markers. *N* = 3 independent experiments. **f** FLAG IP was performed as in Fig. 2f using the indicated FLAG-tagged constructs as bait. *N* = 3 independent experiments. **g** A schematic of PGRMC1 topology indicating the cargo and RTN3-binding regions. Source data are provided as a Source Data file. See also Fig. S2.

As a putative cargo receptor (Fig. 2f), we reasoned that PGRMC1 should use its luminal domain to capture the mutant prohormone contained within the ER lumen. With this in mind, we first sought to validate the topology of PGRMC1. Multiple reports have proposed PGRMC1 to be a single-pass type-II transmembrane protein with a cytosolic NTD and an ER luminal C-terminal domain (CTD) (Fig. 3a^35–38^), although numerous other studies support a reverse topology with the NTD facing the ER lumen^39,40^. To firmly establish PGRMC1 topology in 293 T cells, microsomes from mechanically disrupted cells were exposed to a low concentration of trypsin, and the digested samples subjected to SDS-PAGE. As a positive control for this assay, we first analyzed the topology of endogenous DNAJB12, which has been shown to have a type-II transmembrane protein topology in the ER membrane^41^. Specifically, DNAJB12 contains a large 243 amino acid NTD facing the cytosol and a smaller 110 amino acid CTD within the ER lumen^41^. Whereas Triton X-100 solubilization resulted in subsequent complete proteolytic degradation of full-length DNAJB12 (Fig. S2a, lane 3), a protected tryptic fragment of ~13–14 kDa (a size expected of the 110 amino acids of the DNAJB12 CTD) remained after digestion of microsomes in the absence of detergent (Fig. S2a, lane 2), indicating protection by the ER membrane.

In parallel, we followed PGRMC1 by immunoblotting with antibodies recognizing either the NTD or CTD. We observed that α-PGRMC1-CTD detected a faster migrating endogenous species (Fig. 3b top) corresponding to trypsin-truncated PGRMC1, whereas α-PGRMC1-NTD did not detect any band after trypsin treatment (Fig. 3b bottom). Similar to that for DNAJB12, all PGRMC1 protein was lost upon trypsin digestion after Triton X-100 solubilization (Fig. 3b lane 3). Additionally, when PGRMC1 with a FLAG-tagged cytosolic NTD (FLAG-PGRMC1) was expressed in a PGRMC1 knockout (KO) cell line, α-PGRMC1-CTD immunoblotting of trypsin-digested WCL again detected a truncated PGRMC1 species (Fig. 3c top) whereas α-FLAG did not detect any band (Fig. 3c bottom). Thus in 293 T cells, the PGRMC1-NTD is exposed to the cytosol (lost upon tryptic cleavage) and the CTD is protected within the ER lumen^35^, and this same type-II topology is observed for both endogenous untagged and FLAG-tagged PGRMC1.

We also used the protease protection assay to clarify the cellular localization of PGRMC1. Intact HEK 293 T cells were treated with protease, and the resulting cellular extract subjected to SDS-PAGE and immunoblotting using an antibody against the N-terminal domain (NTD) of PGRMC1. We reasoned that if plasma membrane-localized PGRMC1 has a type I membrane protein topology (C-terminus in the cytosol and N-terminus in the lumen), we expect the N-terminus of any plasma membrane-localized PGRMC1 to be extracellular, and therefore completely exposed to protease. However, if PGRMC1 displays a type-II membrane protein topology (C-terminus in the lumen and N-terminus in the cytosol), then the C-terminus of any plasma membrane-localized PGRMC1 should be extracellular, and the large C-terminus of PGRMC1 would be degraded by protease, while the short NTD and transmembrane sequence would be protected. In that case, blotting of cells using the PGRMC1-NTD antibody would show a significant mobility shift of any plasma membrane-localized PGRMC1 due to C-terminal cleavage. We found no decrease in PGRMC1-NTD signal upon protease treatment (except when detergent is present), and no mobility shift is observed (Fig. S2b). Therefore, a negligible level of PGRMC1 is at the cell surface.

Next, we generated PGRMC2 with a FLAG-tagged cytosolic NTD (FLAG-PGRMC2), a PGRMC1 ortholog bearing a distinct CTD (Fig. 3a). Importantly, unlike PGRMC1, IP of FLAG-PGRMC2 did not pull down C28F-POMC (Fig. 3d top, lane 5 vs 1). We then swapped the luminal CTD of PGRMC2 with that of PGRMC1 (generating a construct called FLAG-H2, Fig. 3a), and indeed, when expressed at comparable levels to FLAG-PGRMC2, FLAG-H2 co-IP’d C28F-POMC-Myc (Fig. 3d top, lanes 3 vs 5). Additionally, a hybrid protein harboring the cytosolic NTD of PGRMC2 but containing the transmembrane and luminal CTD of PGRMC1 could still engage the cargo (Fig. 3d top, lane 2). However, a hybrid protein that harbors the transmembrane and CTD of PGRMC2 with the NTD of PGRMC1 (FLAG-H3, Fig. 3a) could not associate with the substrate (Fig. 3d, lane 4). Together, these results firmly demonstrate that the luminal CTD of PGRMC1 is both necessary and sufficient for the luminal recruitment of ER-phagic cargo.

Because PGRMC1 can physically complex with mutant POMC by co-immunoprecipitation, we next asked if PGRMC1 can bind directly to misfolded POMC. To this end, we used commercially available POMC-6xHis purified from E*. coli* (Fig. 3e, lane 1), FLAG-PGRMC1 purified from HEK 293 T cells (Fig. 3e, lane 3), and the control protein bovine serum albumin (BSA) (Fig. 3e, lane 2). We note that although POMC-6xHis is derived from the WT sequence of POMC, its expression in the bacterial cytosol precludes glycosylation (Fig. S2c, top panel) and ER-mediated folding. Importantly, purified POMC-6xHis pulled down FLAG-PGRMC1 but not BSA (Fig. 3e, compare lane 5 to 4), indicating that PGRMC1 can directly bind to misfolded POMC.

To further analyze the PGRMC1-mutant POMC interaction, we sought to determine the region within the PGRMC1-CTD that binds to cargo. Because PGRMC1 but not PGRMC2 binds to C28F-POMC (Fig. 3d), we aligned the amino acid sequences of PGRMC1 and PGRMC2 to identify divergent regions (Fig. S2d). We observed that although most of the C-terminal regions of PGRMC1 and PGRMC2 are identical, lesser conservation is found at the extreme C-terminus. Accordingly, we generated a truncated PGRMC1 construct containing amino acids 1–170 of PGRMC1 but lacking the final 25 amino acids of this protein (FLAG-PGRMC1_1–170_). Strikingly, in contrast to full-length FLAG-PGRMC1, FLAG-PGRMC1_1–170_ cannot interact with C28F-POMC in cells (Fig. 3f), suggesting that the PGRMC1 cargo-binding domain requires its extreme C-terminus. It remains possible that other regions of the PGRMC1 C-terminus assist in substrate interaction in tandem with the region downstream of residue 170.

We next asked whether cargo binding by the PGRMC1 luminal CTD is sufficient to promote cargo degradation. Remarkably, whereas full-length FLAG-PGRMC1 expression confers degradation of C28F-POMC-Myc in PGRMC1 KD cells (Fig. S2e, lane 3 vs 2; quantified in Fig. S2f), expression of neither FLAG-H1 nor FLAG-H2 (both of which can bind the luminal cargo) could promote C28F-POMC-Myc degradation (Fig. S2e; quantified in Fig. S2f). Thus, cargo association with the PGRMC1 luminal CTD is not itself sufficient to properly dispose of the cargo. [As expected, FLAG-H3 or FLAG-PGRMC2—neither of which can even bind the cargo—also cannot promote degradation of C28F-POMC-Myc (Fig. S2e; quantified in Fig. S2f)].

These results prompted us to examine the role of the cytosolic NTD of PGRMC1 during ER-phagic degradation. We found that a FLAG-PGRMC1 construct lacking the NTD (FLAG-PGRMC1ΔN, Fig. 3a) binds to cargo more efficiently than full-length FLAG-PGRMC1 (Fig. S2g; quantified in Fig. S2h), yet this truncated PGRMC1 protein could not functionally restore cargo degradation in PGRMC1 KD cells (Fig. S2i, compare lane 4 to 3; quantified in Fig. 2j). These results suggest that both the NTD and CTD are required for PGRMC1 to serve as a functional ER-phagy cargo receptor (see Discussion).

In addition to identifying the cargo-binding region in PGRMC1, we sought to clarify how PGRMC1 interacts with RTN3. For this, we compared the interaction between FLAG-RTN3A and HA-tagged full-length PGRMC1 (HA-PGRMC1), PGRMC1 deleted for its cytosolic N-terminal region (HA-PGRMC1ΔN), or PGRMC1 deleted for its ER luminal C-terminal region (HA-PGRMC1ΔC). We found that each PGRMC1 variant can be co-immunoprecipitated by FLAG-RTN3A (Fig. S2k). Because the only shared region in PGRMC1ΔN and PGRMC1ΔC is the transmembrane domain of PGRMC1, our results are consistent with the possibility that PGRMC1 interacts with RTN3 via its transmembrane region. In sum, our analyses indicate that the extreme C-terminus of PGRMC1 is required for binding to misfolded cargo, while the transmembrane region of PGRMC1 interacts with RTN3 (Fig. 3g).

### PGRMC1 promotes degradation of select proinsulin mutants

We previously found that RTN3 also triggers ER-phagic clearance of the classic mutant *Akita* proinsulin that causes the diabetes syndrome known as MIDY^30^. *Akita* in fact represents one of ~30 different missense mutations found in the human *INS*-gene responsible for this diabetic condition^26,42^.

As PGRMC1 is part of the RTN3-dependent ER-phagy targeting complex, we screened for the steady-state level of seven different Myc-tagged MIDY mutants under PGRMC1 KD or RTN3 KD (the KD levels shown in Fig. S3a, b, respectively, and the specific mutations within proinsulin are depicted in Fig. S3c). The levels of individual MIDY mutants in the soluble and insoluble fractions were assessed identically to that described above for C28F-POMC. We defined mutant proinsulin cargos as PGRMC1 dependent if they accumulated (in either the soluble or insoluble fraction) ≥ 2 fold under PGRMC1 KD conditions.

**Fig. 4:**
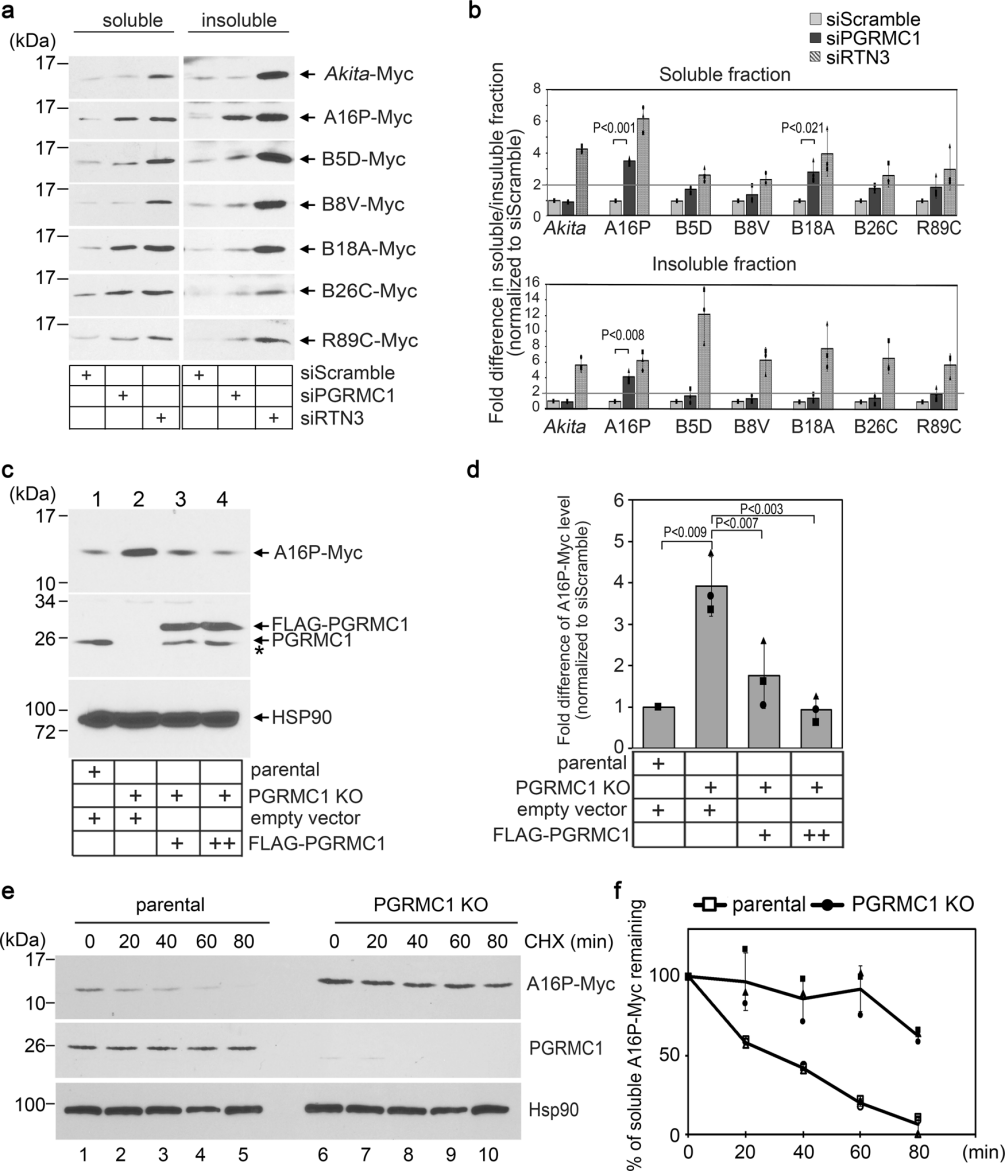
PGRMC1 promotes degradation of select proinsulin mutants. **a** HEK 293 T cells expressing the indicated proinsulin mutant were treated with scrambled, PGRMC1, or RTN3 siRNA. Soluble and insoluble fractions were collected as in Fig. 2a, and the samples were subjected to SDS-PAGE and immunoblotted using a Myc antibody. *N* = 3 independent experiments. **b** Quantification of proinsulin mutant level from **a** relative to the scrambled siRNA. Data are represented as mean ± SD. *N* = 3 independent experiments. One-tailed Standard Student’s *t*-test was used to determine statistical significance. **c** HEK 293 T parental and PGRMC1 KO cells expressing A16P-Myc and co-transfected with either an empty vector or HA-PGRMC1 were lysed and subject to SDS-PAGE and immunoblotted as indicated. *The band below the FLAG-PGRMC1 band in lanes 3 and 4 likely represents degraded FLAG-PGRMC1. *N* = 3 independent experiments. **d** Quantification of the A16P-Myc level from **c** relative to parental cells (lane 1). Data are represented as mean ± SD. *N* = 3 independent experiments. One-tailed Standard Student’s *t*-test was used to determine statistical significance. **e** HEK 293 T parental and PGRMC1 KO cells expressing A16P-Myc were treated with cycloheximide for 0, 20, 40, 60, 80 min. Cells were lysed and subjected to SDS-PAGE and immunoblotted as indicated. *N* = 3 independent experiments. **f** Quantification of the A16P-Myc level from **e** relative to t = 0 for either the parental or PGRMC1 KO cells. Data are represented as mean ± SD. *N* = 3 independent experiments. One-tailed Standard Student’s *t*-test was used to determine statistical significance. *P ≤ 0.05; **P ≤ 0.005. Source data are provided as a Source Data file. See also Fig. S3.

Using this criterion, the level of the L105(A16)P-Myc MIDY mutant (referred to simply as A16P-Myc) in both the soluble and insoluble fractions increased by 3–4 fold under PGRMC1 KD (Fig. 4a; quantified in black bars of Fig. 4b). Similarly, the level of soluble V42(B18)A-Myc (referred to as B18A-Myc) increased by three fold due to PGRMC1 depletion (Fig. 4a; quantified in Fig. 4b). By contrast, the level of *Akita*-Myc was unaffected under PGRMC1 KD, while the remaining proinsulin mutants were only modestly affected (Fig. 4a; quantified in Fig. 4b). In contrast, RTN3 KD led to accumulation of all the MIDY mutants in both fractions (Fig. 4a; quantified in Fig. 4b). Thus, PGRMC1 triggers the clearance of a selective subset of MIDY proinsulin mutants, while RTN3 is more broadly involved in the degradative removal of these mutants.

To further support the idea that PGRMC1 acts to degrade selected proinsulin mutants, we generated a PGRMC1 knockout (KO) HEK293T cell line (Fig. 4c second panel, lane 2 vs 1). Not surprisingly, the level of A16P-Myc in the soluble fraction robustly accumulated in PGRMC1 KO cells (Fig. 4c top, lane 2 vs 1; quantified in Fig. 4d), consistent with the PGRMC1 KD phenotype (Fig. 4a, b). In a rescue experiment, expression of increasing concentrations of FLAG-PGRMC1 in the KO cells decreased accumulation of soluble A16P-Myc (Fig. 4c top, lanes 3, 4 vs 2; quantified in Fig. 4d). A CHX chase further revealed that degradation of A16P-Myc in the soluble fraction was considerably inhibited in PGRMC1 KO cells (Fig. 4e; quantified in Fig. 4f), unambiguously establishing that the expression of PGRMC1 is linked to the degradative clearance of this mutant proinsulin.

### Loss of PGRMC1 causes mutant proinsulin accumulation at a defined ER subdomain

**Fig. 5:**
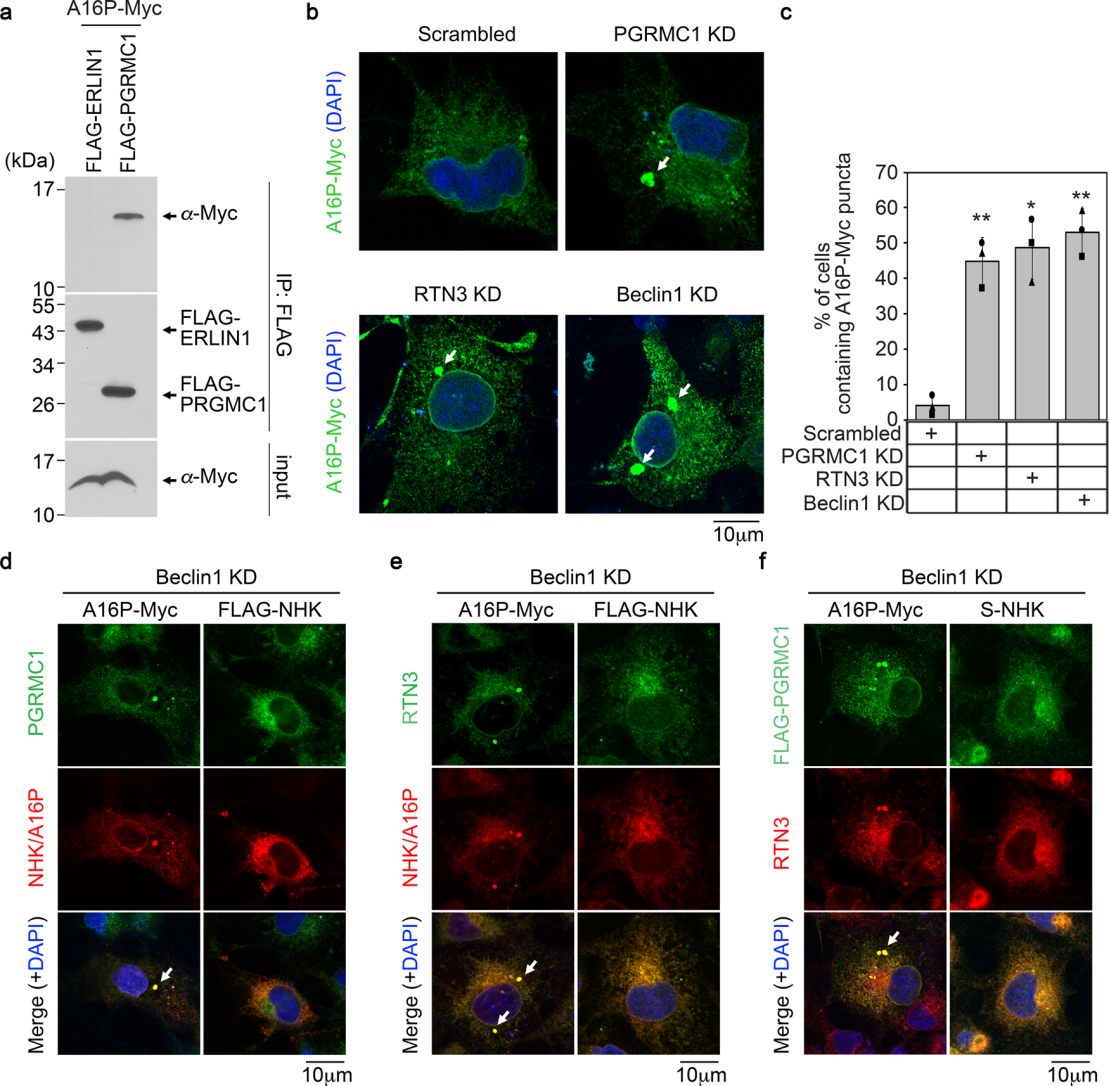
Loss of PGRMC1 causes mutant proinsulin accumulation at a defined ER subdomain. **a** HEK 293 T cells expressing A16P-Myc and either FLAG-ERLIN1 or FLAG-PGRMC1 were subjected to FLAG IP as in Fig. 2f. *N* = 3 independent experiments. **b** COS-7 cells expressing A16P-Myc and co-transfected with either scrambled, PGRMC1, RTN3, or Beclin1 siRNA were fixed, stained, and imaged by confocal microscopy. *N* = 3 independent experiments. **c** Quantification of **b** in which A16P-Myc puncta that are >0.2 μm are scored; each condition represents at least 50 cells per experiment, *N* = 3 independent experiments. Data are represented as mean ± SD. One-tailed Standard Student’s *t*-test was used to determine statistical significance. From left to right, corresponding *p*-values are: <0.002, <0.005, <0.002. **d–f** COS-7 cells expressing A16P-Myc or FLAG-NHK were treated with siRNA against Beclin1, fixed, stained, and imaged by confocal microscopy. *N* = 3 independent experiments. *P ≤ 0.05; **P ≤ 0.005. Source data are provided as a Source Data file. See also Fig. S4.

IP of FLAG-PGRMC1 co-IP’d A16P-Myc (Fig. 5a), indicating that PGRMC1 (but not FLAG-ERLIN1) associates with the cargo. We previously found that when ER-phagy is unable to proceed, mutant proinsulin accumulates in large puncta at select ER subdomains^30,43^. Thus, we asked if loss of PGRMC1 causes A16P-Myc to accumulate in similar puncta. (To better visualize the fate of A16P in the ER, we used COS-7 cells.) Indeed, PGRMC1 KD cells revealed distinct A16P-positive puncta (> 0.2 μm) in the ER (Fig. 5b; quantified in Fig. 5c), an observation that parallels biochemically detectable accumulation of the ER-phagy substrate (Fis. 4a, c). By contrast, under PGRMC1 KD, *Akita*-Myc did not accumulate in distinct puncta (Fig. S4a), consistent with the fact that it is not a PGRMC1-dependent substrate (Fig. 4a, b). A16P-positive puncta also accumulated under RTN3 KD (Fig. 5b; quantified in Fig. 5c), in agreement with the finding that RTN3 depletion increased the A16P level (Fig. 4a, b). Together, these data suggest that depletion of the PGRMC1-RTN3 targeting complex causes the A16P-mutant proinsulin to accumulate within an ER subdomain.

To confirm that A16P is degraded by autophagy, we found that silencing Beclin1 robustly increased A16P levels in both soluble and insoluble fractions (Fig. S4b; quantified in Fig. S4c). Additionally, although Beclin1 KD did not increase the protein levels of endogenous RTN3 or PGRMC1 (Fig. S4b), Beclin1 KD did lead to accumulation of A16P-positive puncta (Fig. 5b; quantified in Fig. 5c). Moreover, although PGRMC1 did not accumulate in any visible puncta in control cells expressing A16P (or the irrelevant misfolded protein NHK) (Fig. S4d), PGRMC1 clearly accumulated in A16P-positive puncta under Beclin1 KD (Fig. 5d, first column). PGRMC1 did not appear to accumulate in visible puncta even under Beclin1 KD in the absence of ER-phagy substrate (Fig. 5d, second column); NHK was used as a control because while it is a misfolded protein, it does not enter the ER-phagy pathway and is instead degraded exclusively via ERAD^44^. RTN3 also accumulated in A16P-positive puncta in Beclin1 KD cells (Fig. 5e, first column), but neither in control cells expressing A16P (Fig. S4e) nor in Beclin1 KD cells in the absence of ER-phagy substrate (Fig. 5e, second column). Not surprisingly, as PGRMC1 interacts with RTN3, both membrane proteins accumulate in the same puncta in A16P-expressing Beclin1 KD cells (Fig. 5f, first column) but neither accumulate in large puncta in A16P-expressing control cells (Fig. S4f) nor in Beclin1 KD cells in the absence of ER-phagy substrate (Fig. 5f, second column). These results support the idea that this proinsulin mutant is degraded by macroautophagy, but when the PGRMC1-RTN3 membrane complex cannot effectively deliver the cargo to the autophagy machinery, the ER-phagy targeting complex becomes blocked along with the cargo.

### PGRMC1 selects small cargos for degradation

**Fig. 6:**
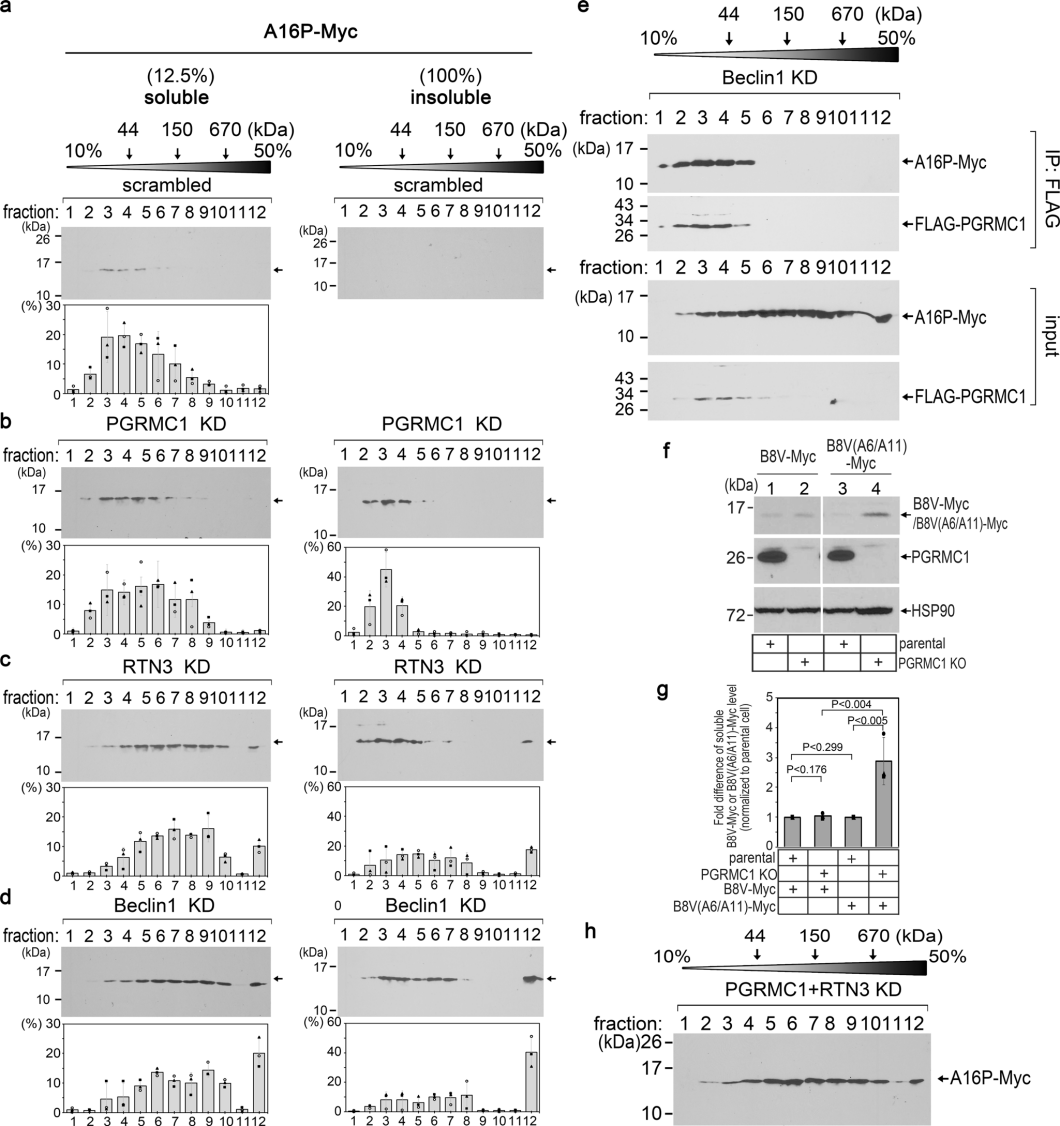
PGRMC1 selects small cargos for degradation. **a–d** HEK 293 T cells expressing A16P-Myc were transfected with scrambled, PGRMC1, RTN3 or Beclin1 siRNA. The soluble and insoluble fractions were collected, layered over a discontinuous 10–50% sucrose gradient, and centrifuged. Each fraction was collected, subjected to SDS-PAGE, and immunoblotted as indicated. Quantification of three separate replicates for each sucrose gradient is shown below each panel. Data are represented as mean ± SD. **e** HEK 293 T cells expressing A16P-Myc and FLAG-PGRMC1 were transfected with Beclin1 siRNA. FLAG IP was performed as in Fig. 2f, and the bound material was eluted using 3xFLAG peptide. Whole-cell lysate (input) and the precipitated material were subject to sucrose gradient analysis followed by SDS-PAGE and immunoblotting as described in **a–d**. *N* = 3 independent experiments. **f** HEK 293 T parental or PGRMC1 KO cells expressing B8V-Myc or B8V(A6/A11)-Myc were lysed, subject to SDS-PAGE, and immunoblotted as indicated. *N* = 3 independent experiments. **g** Quantification of mutant proinsulin from **f** (top panels) relative to parental cells. Data are represented as mean ± SD. *N* = 3 independent experiments. One-tailed Standard Student’s *t*-test was used to determine statistical significance. **h** As in **a–d** except cells were co-transfected with PGRMC1 and Beclin1 siRNAs. *N* = 3 independent experiments. *P ≤ 0.05; **P ≤ 0.005. Source data are provided as a Source Data file. See also Fig. S5.

MIDY proinsulin mutants are known to form protein complexes that are wide-ranging in size, even though monomeric proinsulin is only ~10 kDa. We therefore probed the size of A16P that is recognized by PGRMC1. The level of (soluble and insoluble) undegraded A16P increased under PGRMC1 KD (Fig. 4a, b; Fig. S4b, c), and we reasoned that the size of these accumulated A16P species likely reflects the cargo size normally recognized by PGRMC1. To evaluate the size of A16P complexes, both soluble and insoluble fractions derived from A16P-expressing cells were subjected to sucrose gradient sedimentation, and the individual fractions assessed by reducing SDS-PAGE followed by immunoblotting. In control cells, the majority of soluble A16P was found in fractions 3–5 corresponding to a molecular weight (MW) of less than 70–80 kDa (Fig. 6a soluble fraction; quantified in the graph below) while no A16P was insoluble (Fig. 6a insoluble). Based on size, these complexes amount to ≤ 8 copies of A16P protein per complex. In PGRMC1 KD cells, most soluble A16P was found in fractions 3–7 that still correspond to a MW of ≤ 150 kDa (Fig. 6b soluble fraction; quantified in graph below) and essentially all insoluble A16P was confined to fractions 2–4 corresponding to complexes whose proinsulin component involves a MW ≤ 44 kDa (Fig. 6b, insoluble fraction; quantified in graph below). Thus, our analysis suggests that PGRMC1 recognizes species whose proinsulin-A16P component is ≤ 150 kDa.

By contrast, the size of A16P (in both soluble and insoluble fractions) was appreciably larger under RTN3 KD, with the A16P species appearing in denser fractions including fraction 12 that contains megadalton-sized protein complexes (Fig. 6c soluble and insoluble fractions; quantified in the graph below). Similar to RTN3 KD, under Beclin1 KD, A16P (in both soluble and insoluble fractions) was also found in denser fractions including fraction 12 (Fig. 6d soluble and insoluble fractions; quantified in the graph below). It should be noted that because insoluble A16P was found in the dense fractions (fractions 5–7 and 12) under Beclin1 KD, the absence of insoluble A16P in these fractions under PGRMC1 KD cannot be due to SDS-induced disassembly of A16P complexes. Thus, our results suggest that PGRMC1 recognizes A16P as a relatively small-sized low MW (LMW) cargo (<150 kDa). This pool of smaller complexes could then either enlarge into larger complexes when PGRMC1 releases its cargo, or a separate pool of larger-sized (>150 kDa) complexes could use a parallel PGRMC1-independent pathway for RTN3-mediated targeting to ER-phagy (these two possibilities considered further, below).

As a second strategy, we directly examined the size of A16P that bound to PGRMC1. In this experiment, FLAG-PGRMC1 was IP’d from Beclin1 KD cells expressing A16P-Myc, the FLAG-PGRMC1 and its associated A16P-Myc eluted by excess FLAG peptide, and the size of these proteins analyzed by sucrose gradient sedimentation [cells were depleted of Beclin1 because Beclin1 KD causes accumulation of both the LMW and HMW species of A16P (Fig. 6d)]. Strikingly, we found that only the LMW species of A16P-Myc were co-precipitated with FLAG-PGRMC1 (Fig. 6e top) despite the availability of both forms of A16P-Myc (Fig. 6e, input). This result independently demonstrates that PGRMC1 selectively engages LMW cargos.

As yet a third strategy, we sought to convert a HMW cargo (not recognized by PGRMC1) into a LMW cargo in order to test if it could become a PGRMC1-dependent substrate. To this end, we found that the steady-state level of the B8V-Myc mutant proinsulin was unaffected under PGRMC1 KD (Fig. 4a, b). By nonreducing SDS-PAGE, most B8V-Myc is found in HMW protein complexes (Fig. S5a). These HMW complexes are likely polymers of monomeric B8V that are linked by non-native intermolecular disulfide bonds^45^. We have previously found that mutating two select proinsulin Cys residues [Cys(A6) and Cys(A11)] in the background of the B8V mutation (and other MIDY mutations) decreased the formation of intermolecular disulfide bonds, thereby blocking HMW complex formation and leading to more LMW species^46^ Reproduced here, the “B8V(A6/A11)” mutant forms lower MW disulfide-linked complexes when compared to B8V alone (nonreducing SDS-PAGE, Fig. S5a). Importantly, whereas soluble B8V-Myc did not accumulate in PGRMC1 KO cells (Fig. 6f lane 2 vs 1; quantified in Fig. 6g), the level of soluble B8V(A6/A11)-Myc markedly increased due to loss of PGRMC1 (Fig. 6f lane 4 vs 3; quantified in Fig. 6g). This result strongly supports that PGRMC1 initiates the targeting of LMW cargos during ER-phagic clearance of misfolded substrates.

Because B18A-proinsulin is also a PGRMC1-dependent cargo (Fig. 4a, b), we examined the size of the accumulated B18A species under PGRMC1 KD and found a similar pattern as A16P: soluble B18A was found in fractions 3–8 corresponding to a MW of less than 180 kDa (Fig. S5b top), whereas this mutant proinsulin was found in denser fractions corresponding to HMW proteins under KD of RTN3 (Fig. S5b middle) or Beclin1 (Fig. S5b bottom). Moreover, we found that in the PGRMC1 KD cells, the majority of insoluble C28F-POMC (Fig. 2a, c) was found in fractions 2–4, corresponding to a MW of less than 44 kDa (Fig. S5c). Altogether, these findings further support the idea that PGRMC1 has a substrate size limitation when functioning as a receptor of luminal cargo for ER-phagy.

It seems surprising that when ER-phagy is impaired, C28F-POMC forms LMW complexes yet a pool of these LMW complexes is still detergent-insoluble. Nevertheless, we found that C28F-POMC in the detergent-insoluble fraction is more resistant to digestion by increasing protease concentrations when compared to the soluble fraction (Fig. S5d; quantified in S5e). This indicates that insoluble mutant POMC is in a physical state which is biochemically distinct from its soluble counterpart. Given that when ER-phagy is compromised, mutant POMC becomes detergent-insoluble (Fig. 2a), is protease resistant (Fig. S5d, e), and accumulates in bright puncta (Fig. S1F), we propose that insoluble mutant POMC exists in a different state than soluble mutant POMC, despite being in small protein complexes.

Although degradation of *Akita* depends on RTN3 (Fig. 4a, b) and Beclin1-dependent autophagy^30^, this mutant proinsulin does not use PGRMC1 for clearance (Fig. 4a, b). One possibility is that most *Akita* proinsulin may intrinsically form much larger protein complexes than PGRMC1 is able to recognize. In control cells, soluble *Akita* was found throughout the sucrose gradient including the denser fractions 6–12 (Fig. S5f, top panel), whereas most A16P was found in fractions 3–5 (Fig. 6a, top panel), suggesting that *Akita* exists in larger protein complexes than A16P. Additionally, under RTN3 or Beclin1 KD, the soluble *Akita* proinsulin dramatically shifted its distribution in the sucrose gradient (Fig. S5f*left*) and insoluble *Akita* appeared exclusively in the densest fraction (Fig. S5f*right*), whereas under RTN3 or Beclin1 KD, insoluble A16P remained present throughout the gradient including in lighter fractions 3–7 (Fig. 6c, d). Thus, when prevented from RTN3-dependent ER-phagic degradation, *Akita* accumulates in much higher MW proinsulin complexes, while A16P accumulates in complexes whose proinsulin component remains heterogenous including a large portion of lower MW (<150 kDa) species. These data agree with the idea that PGRMC1 recognizes relatively small cargos to initiate ER-phagic clearance.

We considered that a LMW cargo might mature in size after binding to and release from PGRMC1, thereby converting to a HMW cargo. In this scenario, we expected that if PGRMC1 is depleted, this would block the appearance of HMW A16P-Myc in RTN3 KD cells (Fig. 6c). However, under the condition of PGRMC1/RTN3 double KD, the HMW species of A16P-Myc was still observed (Fig. 6h fractions 7–12). Thus, appearance of HMW A16P in RTN3 KD cells is unlikely to require prior PGRMC1 engagement, but instead may reflect a distinct pool of A16P that utilizes a PGRMC1-independent pathway to gain entry into RTN3-mediated ER-phagy.

### Chemical inactivation of PGRMC1 stabilizes WT and mutant proinsulin, enhancing WT proinsulin export from the ER

**Fig. 7:**
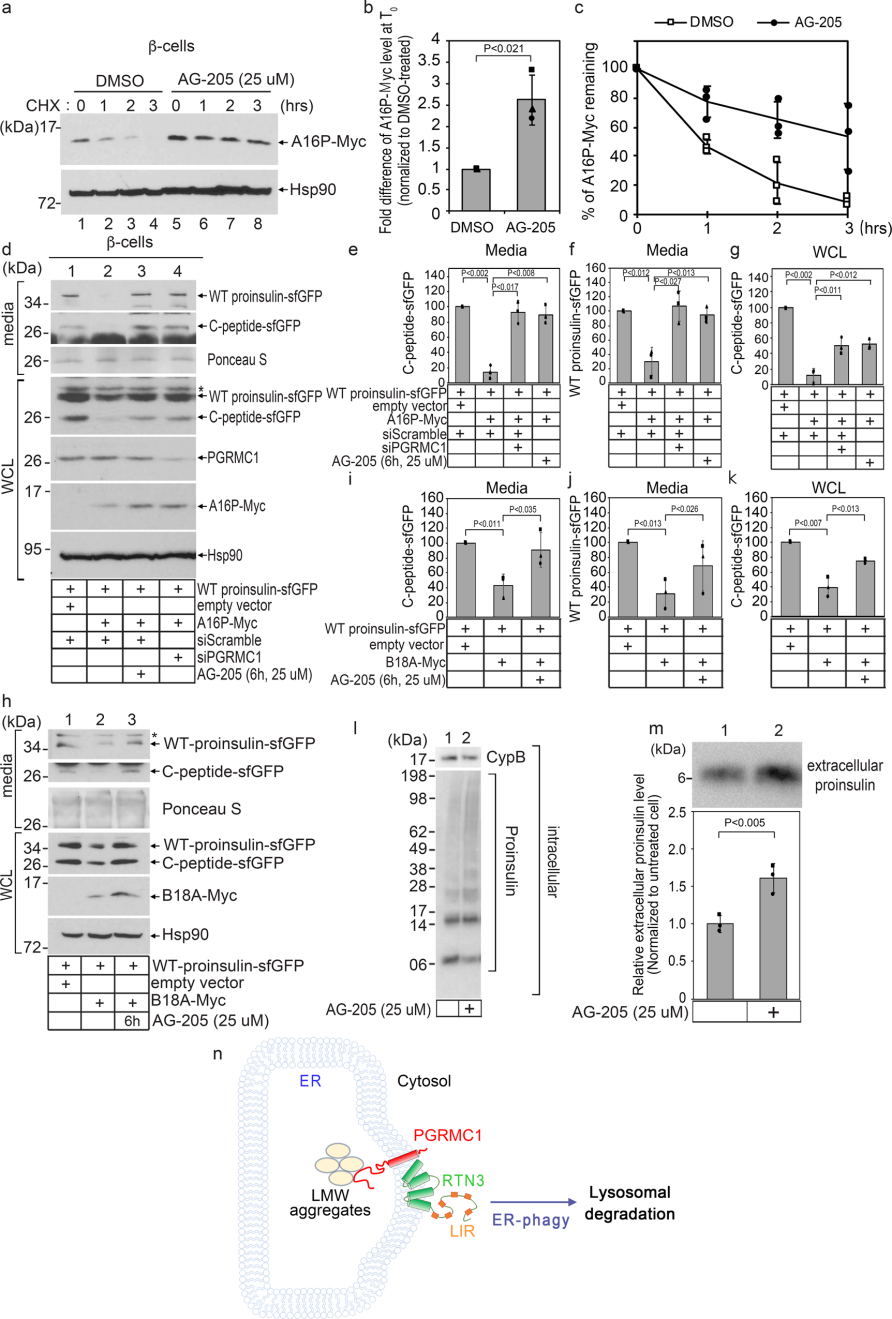
Chemical inactivation of PGRMC1 stabilizes WT and mutant proinsulin, enhancing WT proinsulin export from the ER. **a** INS-832/13 cells expressing A16P-Myc were treated with either DMSO or AG-205 for 2 h, and subsequently treated with cycloheximide for 0, 1, 2, 3 h. Cells were lysed, and the lysate subjected to SDS-PAGE and immunoblotting as indicated. *N* = 3 independent experiments. **b** Quantification of the steady-state A16P-Myc level (at T = 0) from **a** relative to DMSO (lane 5 relative to lane 1). Data are represented as mean ± SD. *N* = 3 independent experiments. One-tailed Standard Student’s *t*-test was used to determine statistical significance. **c** Quantification of the A16P-Myc level relative to T = 0 for DMSO and AG-205 treatment. Data are represented as mean ± SD. *N* = 3 independent experiments. **d** INS-832/13 cells expressing WT proinsulin-sfGFP and co-transfected with either empty vector or A16P-Myc were treated with AG-205 or PGRMC1 siRNA, as indicated. Media and cells (WCL) were collected and subjected to SDS-PAGE and immunoblotted as indicated. *N* = 3 independent experiments. **e** Quantification of C-peptide-sfGFP band intensity from the media fraction from **d** relative to control conditions (lane 1). Data are represented as mean ± SD. *N* = 3 independent experiments. One-tailed Standard Student’s *t*-test was used to determine statistical significance. **f** Quantification of WT proinsulin-sfGFP band intensity from the media fraction from D relative to control conditions (lane 1). Data are represented as mean ± SD. *N* = 3 independent experiments. One-tailed Standard Student’s *t*-test was used to determine statistical significance. **g** Quantification of C-peptide-sfGFP band intensity in the WCL from **d** relative to control conditions (lane 1). Data are represented as mean ± SD. *N* = 3 independent experiments. One-tailed Standard Student’s *t*-test was used to determine statistical significance. **h** As in **d**, except cells were transfected with B18A-Myc. *N* = 3 independent experiments. **i–k** Quantification of **h** as in **e–g**. *N* = 3 independent experiments. **l** Extracts derived from INS1E β-cells treated with or without AG-205 (“intracellular”) were subjected to nonreducing SDS-PAGE and immunoblotted as indicated. *N* = 3 independent experiments. **m** The secreted materials from L (“extracellular”) were subjected to reducing SDS-PAGE and immunoblotted with a proinsulin antibody. Quantification of the secreted proinsulin level. Data are represented as mean ± SD. *N* = 3 independent experiments. One-tailed Standard Student’s *t*-test was used to determine statistical significance. **n** A model depicting the role of PGRMC1 in RTN3-mediated ER-phagy of low-molecular-weight (LMW) protein aggregates. *P ≤ 0.05; **P ≤ 0.005. See also Fig. S6.

In parallel to the genetic KD and KO approaches, we used a chemical strategy to disrupt PGRMC1 function in rat insulinoma INS-832/13 pancreatic β-cells, in order to evaluate the fate of mutant proinsulin in a physiologically relevant cell culture system. By CHX chase in β-cells expressing A16P-Myc, pretreatment of the cells with the PGRMC1 antagonist AG-205^47^ already increased the steady-state level of A16P-Myc prior to CHX addition (T_0_, Fig. 7a top, lane 5 vs 1; quantified in Fig. 7b). Moreover, during CHX chase, AG-205 treatment stabilized A16P-Myc turnover (Fig. 7a, top, lanes 5–8 vs 1–4; quantified in Fig. 7c). These findings further support the notion that diminished PGRMC1 function inhibits degradation of A16P.

In the MIDY diabetic syndrome, mutant proinsulin binds to and entraps WT proinsulin in the ER, preventing ER exit of WT proinsulin and promoting its degradation in parallel^48^ (Fig. S3d). We reasoned that because AG-205 impairs degradation of A16P, WT proinsulin degradation might also be inhibited. If so, persistence of WT proinsulin may provide it with additional opportunity for anterograde transport. In β-cells expressing superfolder GFP-tagged WT proinsulin (where sfGFP is inserted in the C-peptide), unprocessed WT proinsulin-sfGFP and the mature C-peptide-sfGFP were detected intracellularly and in the media (Fig. 7d top; secreted C-peptide-sfGFP quantified in Fig. 7e; secreted WT proinsulin-sfGFP in Fig. 7f). These results indicate that full-length proinsulin is secreted from these cells, and mature C-peptide, generated and stored in intracellular secretory granules, is also secreted. [Of note, unprocessed WT proinsulin or proinsulin-sfGFP has been routinely observed to be recovered in the media bathing β-cell lines in which secretion occurs prior to, or in excess of, conversion to mature insulin and C-peptide^48^].

When A16P was co-expressed in β-cells with WT proinsulin-sfGFP, both intracellular and secreted C-peptide-sfGFP (as well as WT proinsulin-sfGFP) were markedly decreased (Fig. 7d lane 2 vs 1; quantified in Fig. 7e-g). These findings demonstrate that A16P dominantly interferes with the ER exit and secretion of WT proinsulin, consistent with the behavior of other established MIDY mutant proinsulins^26^. Strikingly, when β-cells expressing both WT proinsulin-sfGFP and A16P-Myc were treated with AG-205, the levels of stored intracellular C-peptide were partially restored (Fig. 7d, g) and secretion of C-peptide-sfGFP and WT proinsulin-sfGFP significantly increased (Fig. 7d top, lane 3 vs 2; quantified in Fig. 7e, f). Importantly, KD of PGRMC1 phenocopies treatment with AG-205 (Fig. 7d, lane 4; quantified in Fig. 7e–g), indicating that in the presence of the MIDY mutant A16P, an increased intracellular production of WT insulin and C-peptide-sfGFP, as well as increased secretion of WT proinsulin-sfGFP, accompanies the acute loss of PGRMC1 function. From these findings, it appears that AG-205, (or siRNA)-mediated PGRMC1 inactivation, stabilizes A16P and co-expressed WT proinsulin intracellularly, which culminates in enhanced ER export of WT proinsulin with increased C-peptide and insulin production.

Moreover, we also found that expression of B18A-Myc (another PGRMC1-dependent cargo) in β-cells blunted the secretion of co-expressed C-peptide-sfGFP (Fig. 7hsecond panel, lane 2 vs 1; quantified in Fig. 7i) and WT proinsulin-sfGFP (Fig. 7h, top, lane 2 vs 1; quantified in Fig. 7j), and diminished their intracellular levels (Fig. 7h 4th panel, lane 2 vs 1; quantified in Fig. 7k). However, acute AG-205 treatment reversed these effects (Fig. 7h, top, second, and fourth panels, lane 3 vs 2; quantified Fig. 7i-k). Together, these findings suggest that acute chemical inhibition of PGRMC1 can enhance the export of WT proinsulin in the presence of a co-expressed, dominant-negative MIDY mutant, leading to increased insulin and C-peptide production and secretion. Although we cannot rule out that the phenotypes of AG-205-induced mutant proinsulin stabilization (Fig. 7a) and WT proinsulin export (Fig. 7d) could be caused by off-target effects^49^, we believe this is unlikely since the effects of AG-205 are phenocopied by genetic loss of PGRMC1.

As acute loss of PGRMC1 impairs ER-phagy of selective cargo, it is possible that PGRMC1 inactivation might result in general ER stress activation. To test this, we analyzed the splicing of the unfolded protein response (UPR) transcription factor Xbp1 as a quantitative measurement for ER stress induction^50^. We found that acute loss of PGRMC1 (as well as RTN3 or Beclin1) did not activate ER stress response above control levels (Fig. S6a, lane 1; quantified in Fig. S6b). Adding an artificially imposed ER stress (by tunicamycin treatment, which inhibits N-linked glycosylation leading to glycoprotein misfolding) resulted in a similar acute ER stress response across each condition (Fig. S6a, lane 2; quantified in Fig. S6b), which was restored to normal at 24 h after tunicamycin treatment (Fig. S6a, lane 3; quantified in Fig. S6b). These results support the view that inactivating the PGRMC1-dependent ER-phagy pathway can help to rectify the insulin production defect from WT proinsulin in MIDY, without promoting acute ER stress.

Without any genetic mutation, a fraction of WT proinsulin can also misfold^45^ and misfolded proinsulin has the potential to propagate itself to bystander proinsulin molecules^51^. Thus, we asked if chemical inactivation of PGRMC1 might also provide intracellular WT proinsulin additional opportunity to exit the ER and be secreted. Indeed, in the INS1E β-cell line, proinsulin disulfide-linked complexes appeared less efficiently cleared during a brief exposure to AG-205 (Fig. 7l lane 2 vs 1), accompanied by a modest increase in the level of secreted proinsulin (Fig. 7m lane 2 vs 1; quantified below).

Finally, we examined secretion of newly synthesized WT proinsulin in isolated mouse pancreatic islets radiolabeled with ^35^S-Cys-Met and treated with AG-205 or vehicle alone, and chased for 3 h. Upon IP, SDS-PAGE, and autoradiography, we observed that acute AG-205 treatment increased ER export leading to proinsulin secretion (Fig. S6c), supporting our findings from cells in culture. Thus, it is possible that endogenous PGRMC1 activity may limit export of WT proinsulin, and acutely blocking this target might enhance ER exit of WT proinsulin in the secretory pathway of pancreatic β-cells.

## Discussion

Misfolded proteins in the ER are removed by ERAD and ER-phagy protein quality control pathways. In contrast to ERAD^52^, cellular components that comprise the ER-phagy machinery remain remarkably poorly defined^9,16^. One persistently puzzling question is whether there is an ER membrane receptor that can select and recruit luminally localized misfolded protein cargo for ER-phagy. This physical engagement would represent a decisive step to initiate autophagic destruction of the cargo. Thus far, only six ER membrane proteins—FAM134B, RTN3, ATL3, CCPG1, SEC62, and TEX264—have been shown to function in the targeting of ER-phagy substrates in mammals^17–24^. Except for FAM134B^21^, the mechanism by which these targeting complexes for ER-phagy actually identify their corresponding cargos remains entirely mysterious. Although we previously reported that RTN3 promotes ER-phagic clearance of misfolded prohormones including proinsulin and POMC^30^, RTN3 itself cannot directly recruit luminal cargo because RTN3 itself has no domain within the ER lumen. This has motivated us to search for an RTN3-binding partner that can capture ER cargo on the luminal side.

### PGRMC1 participates in RTN3-dependent ER-phagy

Using an unbiased proteomics approach, we identify PGRMC1 as a bona fide ER transmembrane protein that associates with RTN3. Because the cytosolic domain of RTN3 is not required to bind to PGRMC1, we presume the two hairpin loops of RTN3 within the cytosolic leaflet of the ER mediate interaction with PGRMC1. It remains to be determined whether PGRMC1 might first encounter its substrate proteins within a spatially restricted subregion of the ER. Nevertheless, loss-of-function (KD and KO) clearly reveals that PGRMC1 is required to convey certain mutant prohormones to the lysosome for autophagic degradation via a pathway that relies on RTN3. Hence, PGRMC1 targets the RTN3-dependent ER-phagy of selected substrates (Fig. 7n). It is worth noting that PGRMC1 was previously implicated in macroautophagy^53^.

### The transmembrane protein PGRMC1 is a receptor for misfolded ER luminal cargo

Our biochemical analyses reveal that PGRMC1 associates with mutant cargo proteins. In HEK 293 T cells, protease protection experiments support the postulated topology of PGRMC1 as a type-II transmembrane protein with its NTD oriented towards the cytosol and its CTD in the ER lumen^35–38^, which contrasts the proposed topology of PGRMC1 in other cell types^39,40^. Domain-swapping experiments establish that the CTD of PGRMC1 is both necessary and sufficient for binding to luminally localized cargo. We further pinpointed the extreme C-terminus of PGRMC1 (downstream of residue 170) to be important for cargo binding. Whether other regions of the PGRMC1-CTD are required for cargo binding, or if the extreme C-terminus is sufficient, remains to be determined and will warrant future research. Moreover, the in vitro reconstitution experiments using purified components demonstrate that PGRMC1 can bind directly to cargo. Hence, we conclude that PGRMC1 acts as a cargo-binding receptor within the RTN3 ER-phagy targeting complex. Intriguingly, our mutational analysis also suggests that the cytosolic NTD of PGRMC1 plays a critical role in ER-phagy. One idea is that the NTD domain functions to couple and uncouple from RTN3, or to provide transmembrane signals for the release of cargo from the luminal CTD—such features would allow PGRMC1 itself to avoid RTN3-triggered ER-phagic destruction so that it can release cargo and recycle for additional rounds of cargo capture. Whether PGRMC1 and RTN3 constitute a complete ER-phagy targeting complex, or if there are additional proteins within this complex that are required for substrate recruitment, remains to be determined.

### PGRMC1 cargo recognition is size selective

Beyond simply capturing the cargo, our data demonstrate that PGRMC1 preferentially selects LMW cargos. This conclusion is based on three independent lines of evidence. First, binding studies showed that PGRMC1 interacts with mutant cargos that are <150 kDa, despite the simultaneous availability of HMW cargos (Fig. 6e). Second, when PGRMC1 is depleted, only LMW cargos accumulate, suggesting that PGRMC1 normally recognizes these substrates (Fig. 6a, Fig. S5a, b). Third, conversion of a PGRMC1-independent HMW cargo into a LMW cargo generates a PGRMC1-dependent substrate (Fig. 6f). Thus, the PGRMC1 cargo receptor for ER-phagy is size selective.

Although our analysis indicates that PGRMC1 can directly capture the mutant cargo, it is conceivable that this binding event in cells requires the assistance of partner proteins such as ER-resident chaperones (e.g., BiP). Additionally, it remains to be discovered the precise molecular basis by which PGRMC1 recognition is limited to LMW cargo. One possibility is that recognition features on the substrate (such as hydrophobic patches or non-native disulfide bonds) become progressively masked as LMW cargo polymerizes to HMW cargo—or, as the misfolded protein complex becomes larger, the relative abundance of the required PGRMC1 partner protein declines, thereby eliminating utilization of PGRMC1. In either case, the actual surprise is that recognition and targeting for ER-phagic degradation is not restricted to HMW cargos. Thus, our results suggest that the repertoire of ER-phagy substrates may be more extensive than previously envisioned, opening an avenue for ER quality control.

### PGRMC1 as a potential target to treat insulin-deficient diabetes

Using a physiologically relevant β-cell line, our findings reveal that PGRMC1 promotes degradation of mutant proinsulins known to cause the diabetic syndrome known as MIDY^26^. In this disease, mutant proinsulins disrupt the export of WT proinsulin through dominant-negative interaction within the ER, blocking insulin production^26^. Strikingly, we find that acute inhibition of PGRMC1 function partially restores the export of proinsulin, along with the production of C-peptide and insulin. Thus, blocking ER-phagic proinsulin degradation can enhance the availability of WT proinsulin to escape from the ER.

Mechanistically, how this is achieved is not entirely clear. It is possible that WT proinsulin can disentangle from the mutant proinsulin complex when ER-phagic degradation is decreased or delayed—this might allow the uncoupled WT proinsulin to exit the ER. Alternatively, when the ER-phagy clearance pathway is perturbed, the WT-mutant proinsulin complex remains “trapped” on the ER-phagy receptor targeting complex—this saturates the receptor targeting complex and enables newly synthesized WT proinsulin to bypass this complex and undergo ER exit. Regardless of the precise mechanism, the fact that this druggable target can be specifically chemically inhibited deserves further attention as a means to enhance insulin production for the treatment of diabetes, and for possible use in other, similar diseases.

## Methods

### Cell culture

HEK 293 T (ATCC, Cat# CRL-3216) and COS-7 (ATCC, Cat #CRL-1651) cells were cultured in Dulbecco’s modified Eagle’s medium (DMEM) supplemented with 10% fetal bovine serum and penicillin/streptomycin and incubated at 37 °C and 5% CO_2_. Rat insulinoma INS1 832/13 (Laboratory of Christopher Newgard^54^ and INS1E (Laboratory of Claes B Wolheim^55^ cells were cultured in Rosewell Park Memorial Institute medium (RPMI) 1640 supplemented with 10% fetal bovine serum, 10 mM HEPES (pH 7.5), 1 mM sodium pyruvate, and 50 uM β-mercaptoethanol.

### Antibodies, plasmids, and siRNAs

Antibodies for western blot and immunofluorescence: anti-Myc (Immunology Consultants Laboratory, Cat #RMYC45A, 1:3000 dilution for western blot), anti-Myc (Santa Cruz Biotechnology, Cat# SC-40, 1:100 dilution for immunofluorescence), anti-FLAG (Millipore Sigma, Cat# F7425, 1:3000 dilution for western blot), anti-FLAG (Millipore Sigma, Cat#F3165, 1:3000 dilution for western blot), anti-Hsp90 (Santa Cruz Biotechnology, Cat# sc13119, 1:10000 dilution for western blot), anti-PGRMC1 C-term (laboratory of Dr. Peter Espenshade, 1:3000 dilution for western blot), anti-PGRMC1 N-term (Cell Signaling Technology, Cat #13856 S, 1:2000 dilution for western blot, 1:100 dilution for immunofluorescence), anti-HA (Millipore Sigma, Cat# 11583816001, 1:3000 dilution for western blot, 1:100 dilution for immunofluorescence), anti-RTN3 (Bethyl Laboratories, Cat# A302-­860 A, Boster Biological Technology, Cat# PA2256, 1:1000 dilution for western blot, 1:33 dilution for immunofluorescence), anti-Beclin1 (MBL International, Cat# PD017, 1:3000 dilution for western blot), anti-Hrd1 (Proteintech Group, Cat# 13473-1­AP, 1:3000 dilution for western blot), anti-Lamp1 (Millipore Sigma, Cat# AB2971, 1:2000 dilution for western blot), anti-BiP (Proteintech Group, Cat# ab21685, 1:2000 dilution for western blot), anti-CypB (Thermo Fisher Scientific, Cat# PA1-027A, 1:1000 dilution for western blot), anti-proinsulin (Novus Biologicals, Cat# NB100-73013, 1:3000 dilution for western blot), anti-GFP (Proteintech Group, Cat# 66002-1­Ig, 1:10000 dilution for western blot), anti-HA (Millipore Sigma Cat#11867423001, 1:100 dilution for immunofluorescence), anti-mouse IgG peroxidase (Millipore Sigma, Cat#A4416), 1:3000 dilution for western blot, anti-rabbit IgG peroxidase (Millipore Sigma, Cat#A4914), 1:3000 dilution for western blot, anti-mouse Alexa Fluor 594 (Thermo Fisher, Cat#A-11032, 1:2000 dilution for immunofluorescence), anti-rat Alexa Fluor 488 (Thermo Fisher, Cat#A-11006, 1:2000 dilution for immunofluorescence), anti-mouse Alexa Fluor 488 (Thermo Fisher, Cat#A28175, 1:2000 dilution for immunofluorescence), anti-rabbit Alexa Fluor 488 (Thermo Fisher, Cat #A11008, 1:2000 dilution for immunofluorescence). For siRNA knockdowns: siRTN3: UCAGUGUCAUCAGUGUGGUUUCUUAdTdT, siBeclin1: GGUCUAAGACGUCCAACAAdTdT, siPGRMC1: GGGACAUACAGAAUAGGAAdTdT, siHrd1: GGAGACUGCCACUACAGUUGUdTdT, siScramble: Qiagen All Star Negative Control. Plasmids 3xFLAG-GFP-RTN3C, 3xFLAG-GFP-RTN4A, 3xFLAG-Sec61B, pCMV-FLAG-PGRMC Hybrid 1, pCMV-FLAG-PGRMC Hybrid 2, pCMV-FLAG-PGRMC Hybrid 3, pCMV-FLAG-PGRMCΔN, pCMV-FLAG-PGRMC2 were generated for this study. pTarget­hProL(A16)P­CpepMyc, pTarget­hProC(B5)D­CpepMyc, pTarget­hProC(B8)V­CpepMyc, pTarget­hProC(B18A)A­CpepMyc, pTarget­hProC(B26)C­CpepMyc, pTarget­hProC(R89)C­CpepMyc, and pTarget­hPro-B8V-A6S-A11S­CpepMyc were described previously^46^. pCMV-FLAG-PGRMC1, pCMV-HA-PGRMC1, and sgPGRMC1-Cas9-Puro were gifted by Dr. Peter Espenshade. pcDNA3.1-C28F-FLAG and pcDNA3.1-C28F-myc were gifted by Dr. Ling Qi. pcDNA3.1-FLAG-RTN3A was a gift from Dr. Ivan Dikic. pLJC5-Tmem192-3xHA was acquired from addgene^34^. FLAG-ERLIN1 was described previously^56^, as well as pTarget­hProC(A7)Y­CpepMyc^27^ and pTarget­hProCpepSfGFP^48^. All plasmids constructed for this study have been made available on Addgene. All primers used for cloning and analysis are listed in Supplementary Table 2.

### Plasmid transfection and small interfering RNA (siRNA) knockdown

Cells were transfected with 0.05–2 μg of plasmid DNA using either polyethylenimine (PEI) or Lipofectamine 2000 (Invitrogen, Cat# 11668030) 24 h after seeding. Twenty four hours post-transfection, cells were harvested in PBS supplemented with 10 mM N-ethylmaleimide (NEM). Small interfering RNA (siRNA) was transfected into cells using RNAiMAX (Invitrogen, Cat #13778100) at the time of seeding, and cells were harvested or otherwise treated after 48 h.

### Immunoblotting and immunoprecipitation

For immunoblotting, cells were lysed in 100 μL RIPA buffer supplemented with 10 mM NEM and 1 mM PMSF, incubated on ice for 10 min, and centrifuged. The extract was subjected to SDS-PAGE (reducing conditions were 200 mM DTT) and transferred to either nitrocellulose or PVDF membrane. Membranes were incubated with primary antibodies in TBST with 3% milk or BSA overnight, washed, and incubated with secondary antibodies for 30–60 min prior to exposure using ECL. For endogenous RTN3 IP, three 15 cm plates of HEK 293 T cells were first chemically crosslinked with dithiobis-succinimidyl-propionate (DSP) for 45 min. Then cells were lysed in 1 mL of 1% Triton X-100 in HN buffer (50 mM HEPES pH 7.5, 150 mM NaCl) containing 10 mM NEM and 1 mM PMSF. Cells were then incubated on ice for 10 min and centrifuged. The resulting whole-cell extract was incubated with RTN3 antibody (Boster Biological Technology) at 4 °C overnight. Protein A/G agarose beads (Thermo Fisher Scientific, Cat#20241) were then added, and samples were again incubated at 4 °C for 30 min. Beads were washed with lysis buffer supplemented with NEM and PMSF, and boiled in SDS sample buffer with 100 mM DTT. For IP of FLAG-tagged proteins, HEK 293 T cells transfected with FLAG-tagged plasmid were lysed in 1% deoxy Big CHAP (DBC) or 1% Triton X-100 in NH buffer at 4 °C for 10 minutes and centrifuged at 16,000 × g at 4 °C for 10 min. The supernatant was collected and rotated at 4 °C for 2 h with M2 FLAG-conjugated beads (Millipore Sigma, Cat#A2220). The beads were washed and eluted by 3x FLAG peptide, and subjected to SDS-PAGE, followed by immunoblotting using the indicated antibodies.

### IP-mass spectrometry

HEK 293 T cells were transfected with either 3xFLAG-GFP-Sec61β, 3xFLAG-GFP-RTN3C, or 3xFLAG-GFP-RTN4A. Cells were harvested and immunoprecipitated using FLAG antibodies as described. Precipitated material was eluted using 3xFLAG peptide (0.1 mg/mL) at room temperature for 20 min. The eluate was treated with 10% TCA and incubated on ice for 10 min. The sample was subjected to centrifugation and the precipitated material washed twice in acetone. This precipitate was subject to mass spectrometry analysis at the Taplin Mass Spectrometry Core Facility (Harvard Medical School). The mass spectrometry proteomics data have been deposited to the ProteomeXchange Consortium via the PRIDE^57^ partner repository with the dataset identifier PXD024725 and 10.6019/PXD024725.

### In vitro binding assay

FLAG-PGRMC1 purification: HEK 293 T cells transiently expressing FLAG-PGRMC1 were lysed in buffer containing 150 mM NaCl, 50 mM HEPES, and 1% deoxy Big CHAP with 1 mM PMSF. Samples were pelleted at high speed to remove unbroken cells and debris, and the supernatant was subject to FLAG immunopurification using anti-FLAG affinity gel (Millipore Sigma, Cat#12657) for 2 h at 4 °C. The samples were then washed five times with lysis buffer and FLAG-PGRMC1 was eluted using excess 3xFLAG peptide. POMC-6xHis was purchased from Abcam (Cat# ab108118) and BSA was purchased from Sigma (Millipore Sigma, Cat #A9418). Five hundred nanograms of POMC-6xHis was incubated with either 5 ug of BSA or 5 ug of FLAG-PGRMC1 in the purification buffer for 30 min at 37 °C and IP’d using magnetic NTA beads (Thermo Fisher Scientific, Cat#10103D) for 15 min at 4 °C. Samples were washed five times in the purification buffer and eluted with sample buffer. Samples were run on SDS-PAGE and analyzed by Coomassie blue staining.

### Detergent insolubility assay

As described previously^30^. Briefly, cells were lysed in 100 μL of RIPA buffer (1% Triton, 0.5% sodium deoxycholate, and 1% SDS) with 10 mM NEM and 1 mM PMSF. Samples were incubated on ice for 10 min and centrifuged. The extracted supernatant constitutes the soluble fraction, and the pellet represents the insoluble fraction. The pelleted material was washed with ice-cold PBS (containing NEM and PMSF), then solubilized using a 2% SDS sample buffer for SDS-PAGE and immunoblotting as described.

### Cycloheximide and AG-205 treatments

Following plasmid and siRNA transfection, cells were treated with cycloheximide (VWR, Cat#97064-722) prepared in ethanol at a final concentration of 50 μg/mL for HEK 293 T cells and 100 μg/mL for INS1 832/13 cells for the indicated amount of time. AG-205 (Tocris, Cat#6242) was prepared in DMSO and added to a final concentration of 25 μM. Following treatment, cells were harvested for immunoblot analysis as described.

### Sucrose gradient fractionation

This assay was performed as previously described^43,58^. In brief, cells were lysed in RIPA buffer and the soluble and insoluble material were prepared as described above. Insoluble material was solubilized using RIPA buffer supplemented with 2% SDS. Samples were layered on top of a 10–50% discontinuous sucrose gradient and centrifuged on a Beckman SW50.1 rotor at 100,000 × g for 24 h at 4 °C. Twelve 50 μL fractions were collected and analyzed by SDS-PAGE and immunoblotting.

### CRISPR-Cas9 knockout and plasmid cloning

HEK 293 T cells were transfected with a plasmid encoding PGRMC1-specific gRNA, spyCas9, and the puromycin resistance gene for selection. This plasmid was a gift from the lab of Dr. Peter Espenshade (Johns Hopkins University). Forty eight hours after transfection, cells were treated with 1 μg/mL puromycin for an additional 48 h. Surviving cells were harvested and combined onto one plate to generate a pooled knockout population. Cells were then serially diluted to generate monoclonal PGRMC1 knockout cell lines. Mutant PGRMC1 variants were generated using PCR-based cloning methods.

### Microscopy

COS-7 cells were imaged as described previously^43^. Briefly, COS-7 cells were plated on coverslips and transfected with siRNA or plasmid as described above. Twenty four hours after DNA transfection, cells were fixed and stained with primary antibodies at 4 °C for 1 h. Cells were then washed and incubated with fluorophore-conjugated secondary antibodies for 30 min in the dark at room temperature. Coverslips were mounted with ProLong Gold mounting medium with DAPI (Thermo Fisher, Cat# P36931) and imaged using the Zeiss LSM 780 confocal microscope. FIJI distribution of ImageJ was used for image processing, analyses and quantification.

### Protease protection assay

WT or PGRMC1 knockout HEK 293 T cells were harvested and resuspended in 500 μl of PBS, and homogenized by passing 10 times through a 8 μm clearance ball bearing homogenizer (Isobiotech). Samples were centrifuged at 1200 × g to remove the intact cells. The supernatant fraction was either untreated or treated with 5 μg/ mL of trypsin and 1% Triton X-100 for 15 min on ice. The proteolysis reaction was stopped by addition of 5× SDS sample buffer and subjected to SDS-PAGE and immunoblotted as described above.

### Immunopurification of lysosomes (lyso-IP)

The lyso-IP method was modified from the published protocol^34^. A 15 cm plate of TMEM192-HA expressing HEK293T cells were washed with PBS and then scraped in 1 mL of cold PBS containing 1 mM PMSF. Fifty microliters (equivalent to 5% of the total cells) was reserved for the whole-cell fraction. The remaining cells were homogenized by passing 10 times through a 8 μm clearance ball bearing homogenizer (Isobiotech). The homogenate was then centrifuged at 1000 × g for 2 min at 4 °C and the supernatant containing the cellular organelles including lysosomes was incubated with 100 μL anti-HA magnetic beads on a gentle rotator shaker for 3 min. Magnetically isolated beads and bound lysosomes were washed three times. The bound lysosomes were then lysed in 50 μL extraction buffer (PBS with 1% Triton X-100), and subjected to SDS-PAGE and immunoblotted as described above.

### Secretion assays

Proinsulin trafficking and secretion was measured as described previously^58^. Briefly, INS1 832/13 cells seeded in a six-well plate were transfected with WT-proinsulin-sfGFP and either an empty vector or A16P-Myc in 2 mL of medium. After 8 h, media was removed and replaced with 500 μL of media for 16 h to enrich the secreted material. Cells were treated with AG-205 as described above. Media was collected and centrifuged at 2000 × g for 2 min to remove any cells or debris. 5X SDS-PAGE buffer was added to the resulting supernatant, and the cells were harvested for SDS-PAGE as described previously. Rat pancreatic INS1E β-cells were also treated with 25 uM AG-205 for 3 h. The bathing media were collected and the cells were lysed in RIPA lysis buffer. The cell lysate or media were subject to SDS-PAGE and immunoblotted using proinsulin antibody. Proinsulin band quantification in the reducing gels of lysates and the media were carried out to deduce total intracellular and extracellular proinsulin levels, respectively.

### Deglycosylation assay

HEK 293 T cells expressing C28F-POMC-FLAG were harvested as described previously and treated with Endoglycosidase H (NEB, Cat#P0702S) or PNGase F (NEB, Cat#P0704S) according to the manufacturer’s protocol. Samples were then subject to SDS-PAGE and immunoblotting as described above.

### Pancreatic islet experiments

B6 mouse islets were washed twice with media lacking Cys and Met, ^35^S pulse-labeled for 15 min in DMSO or 25 mM AG-205 containing media lacking Cys and Met, and chased for 3 h in DMSO or 25 mM AG-205 containing complete growth media. Media was collected at chase time. Islets were lysed in RIPA buffer (25 mM Tris, pH 7.5, 100 mM NaCl, 1% Triton X-100, 0.2% deoxycholic acid, 0.1% SDS, 10 mM EDTA) containing 2 mM NEM and a protease inhibitor cocktail. Islet lysates were normalized to trichloroacetic acid-precipitable counts, immunoprecipitated with rabbit anti-mouse proinsulin-1 and -2 antibodies and protein A agarose overnight at 4 °C. Immunoprecipitates were analyzed by nonreducing NuPAGE SDS-PAGE. Gels were fixed and dried, followed by phosphorimaging and autoradiography. University of Michigan’s Institutional Animal Care and Use Committee (IACUC) has approved this study protocol for use of animals to meet the state and federal ethical standards.

### Quantification of immunoblots

All blots were quantified using the NIH ImageJ software and normalized to the indicated loading control. All data are represented as the mean of 3 or more replicates ±SD.

Standard Student’s *t*-test was used to determine statistical significance. *P ≤ 0.05; **P ≤ 0.005; ***P ≤ 0.001.

### Reporting summary

Further information on research design is available in the Nature Research Reporting Summary linked to this article.

## Supplementary information

Supplementary Information

Reporting Summary

## Data Availability

The mass spectrometry proteomics data have been deposited to the ProteomeXchange Consortium via the PRIDE^57^ partner repository with the dataset identifier PXD024725 and 10.6019/PXD024725. All plasmids generated in this study have been made available on Addgene under the deposit #79811 (https://www.addgene.org/Bill_Tsai/). Further information and requests for resources and reagents unique to this study should be directed to and will be fulfilled by the Lead Contact, B.T. (btsai@med.umich.edu). Source data are provided with this paper.

## Source data

Source Data
