# Supplementary material for: PGRMC1 acts as a size-selective cargo receptor to drive ER-phagic clearance of mutant prohormones
Source: Nat Commun. 2021 Oct 13;12:5991. doi: 10.1038/s41467-021-26225-8 (PMC8514460; doi:10.1038/s41467-021-26225-8)
Supplement: Supplementary file 1 — Supplementary Information [file 41467_2021_26225_MOESM1_ESM.pdf]

Figure S1 PGRMC1 is required for lysosomal turnover of C28F-POMC. (Related to Figure 2)

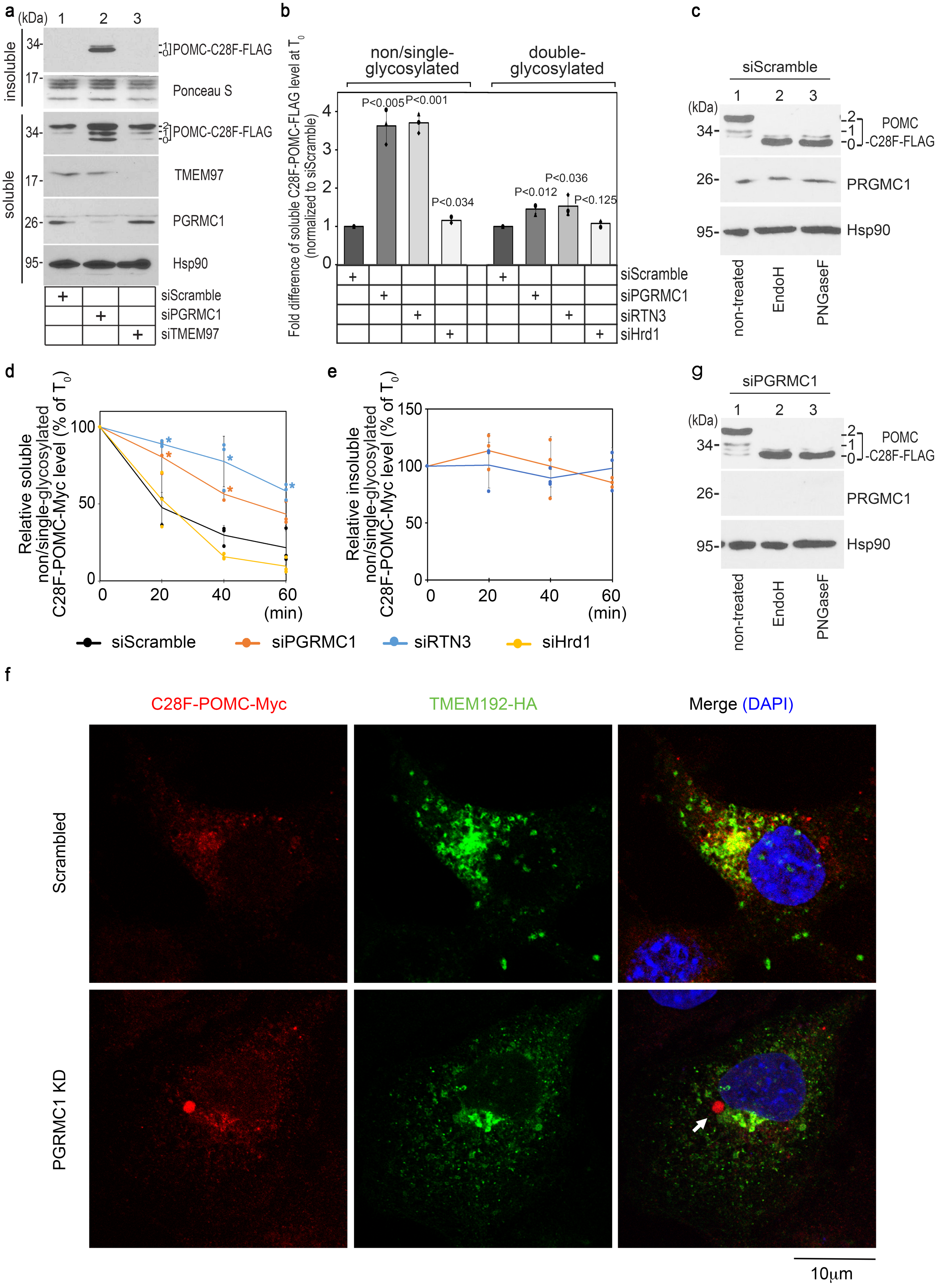

Figure S1. PGRMC1 is required for lysosomal turnover of C28F-POMC. (Related to Figure 2). a HEK 293T cells were treated with the indicated siRNAs and transfected with C28F-POMC-FLAG. Samples were harvested, processed as in Figure 2**a**, and analyzed by SDS-PAGE and immunoblotting. N=3 independent experiments. **b** Quantification of soluble C28F-POMC-FLAG steady-state level (T=0) for each knockdown condition from Figure 2**e**. Non/single-glycosylated refers to the lower two bands of C28F-POMC-FLAG, and double-glycosylated refers to the highest band of C28F-POMC-FLAG. Data are represented as mean ±SD. N=3 independent experiments. One-tailed Standard Student's t test was used to determine statistical significance. **c** HEK 293T cells expressing C28F-POMC-FLAG were lysed and treated with the indicated enzyme for one hour. Samples were subjected to SDS-PAGE and immunoblotted as indicated. N=3 independent experiments. **d-e** Quantification of soluble or insoluble C28F-POMC-FLAG protein levels from Figure 2**e**. Data are represented as mean ±SD. N=3 independent experiments. One-tailed Standard Student's t test was used to determine statistical significance. $^{*}P \leq 0.05$; $^{**}P \leq 0.005$. **f** Confocal imaging of Cos-7 cells treated with the indicated siRNA and expressing C28F-POMC-Myc and TMEM192-HA. Representative images are shown. N=3 independent experiments. **g** HEK 293T cells treated with PGRMC1 siRNA and expressing C28F-POMC-FLAG were lysed and treated with the indicated enzyme for one hour. Samples were subjected to SDS-PAGE and immunoblotted as indicated. N=3 independent experiments. Source data are provided as a Source Data file.

Figure S2 The CTD of PGRMC1 is necessary and sufficient for binding to misfolded POMC.(Related to Figure 3)

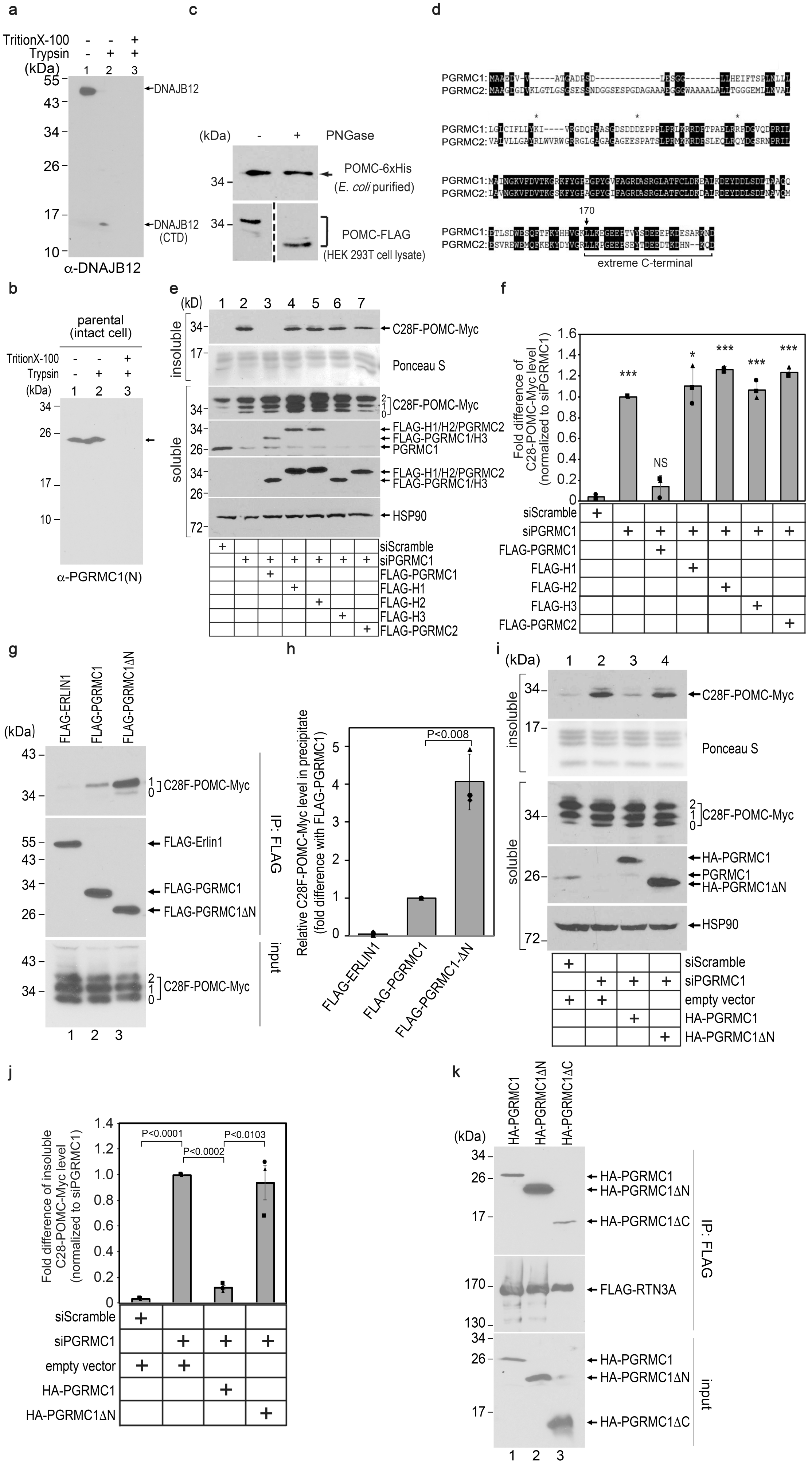

Figure S2. The CTD of PGRMC1 is necessary and sufficient for binding to misfolded POMC. (Related to Figure 3). a Protease protection assay as in Figure 3**b** to confirm the topology of DNAJB12. N=3 independent experiments. B. Intact cells were washed with PBS and treated with protease -/+ Triton X-100, followed by lysis, SDS-PAGE, and immunoblotted as indicated. An arrow shows the position of full-length PGRMC1. N=3 independent experiments. **c** Purified POMC-6xHis or lysate from HEK 293T cells expressing POMC-FLAG were treated with or without PNGase and analyzed by SDS-PAGE and immunoblotting. N=3 independent experiments. **d** Amino acid alignment of PGRMC1 and PGRMC2 with identical residues highlighted in black (Genedoc software). **e** HEK 293T cells expressing C28F-POMC-FLAG were transfected with scramble or PGRMC1 siRNA in addition to the indicated rescue construct. Samples were subjected to SDS-PAGE and immunoblotted as indicated. N=3 independent experiments. **f** Quantification of C28F-POMC-Myc protein level from Figure S2**e**. Data are represented as mean ±SD. N=3 independent experiments. One-tailed Standard Student's t test was used to determine statistical significance. From left to right, corresponding p-values are: <0.001, <0.1424, <0.0052, <0.0001, <0.0001, <0.0002. **g** FLAG IP was performed as in 2**f** using the indicated FLAG-tagged constructs as bait. N=3 independent experiments. **h** Quantification of co-IP'd C28F-POMC-Myc from S3**g** relative to the FLAG-PGRMC1 IP. Data are represented as mean ±SD. N=3 independent experiments. One-tailed Standard Student's t test was used to determine statistical significance. *P ≤ 0.05. **i** HEK 293T cells expressing C28F-POMC-Myc and co-transfected with either an empty vector, HA-PGRMC1, or HA-PGRMC1ΔN were treated with either scrambled or PGRMC1 siRNA, as indicated. Samples were prepared

as in 2**a** and subject to SDS-PAGE and immunoblotting as indicated. N=3 independent experiments. **j** Quantification of insoluble C28F-POMC-Myc protein level from S3**i** normalized to the PGRMC1 siRNA condition (lane 2). Data are represented as mean ±SD. N=3 independent experiments. One-tailed Standard Student's t test was used to determine statistical significance. **k** FLAG IP was performed as in 2**f** using the indicated FLAG-tagged constructs as bait. N=3 independent experiments. Source data are provided as a Source Data file.

Figure S3 A schematic of MIDY proinsulin mutants and the proinsulin maturation pathway. (Related to Figure 4 and Figure 7)

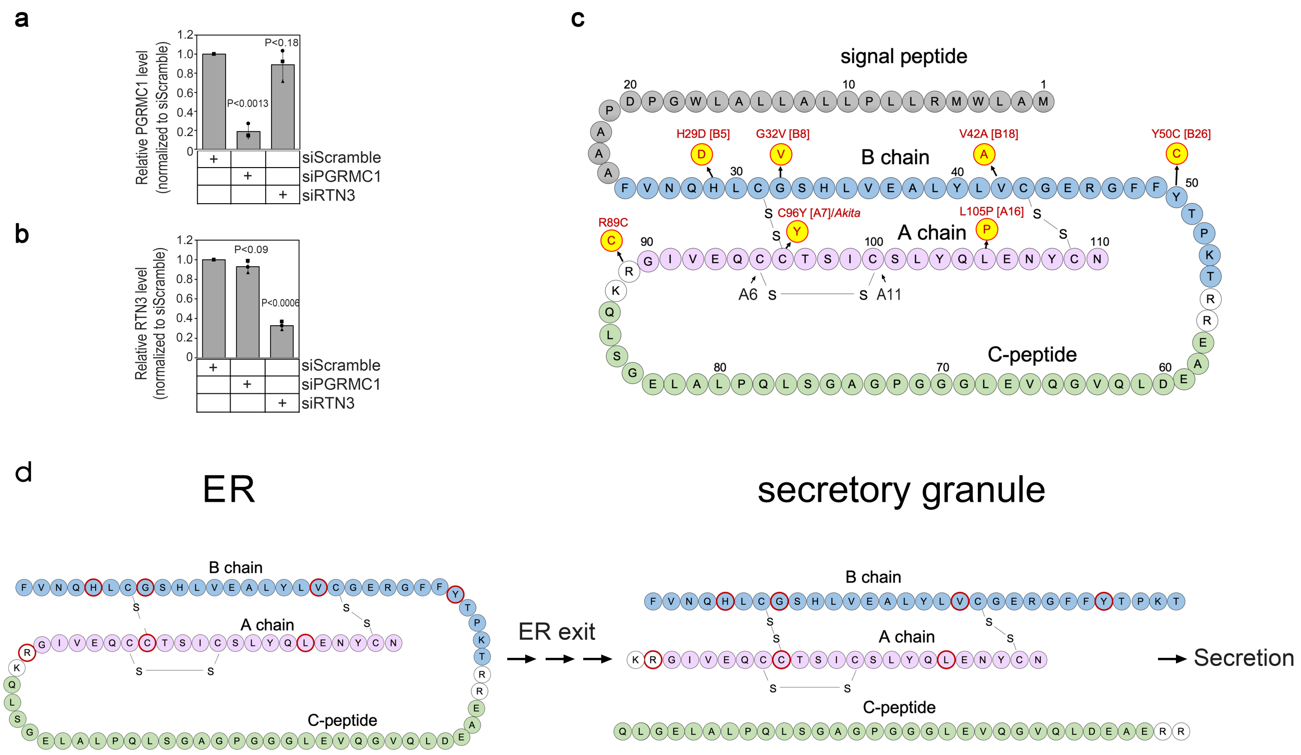

Figure S3. A schematic of MIDY proinsulin mutants and the proinsulin maturation pathway. (Related to Figure 4 and Figure 7). a Quantification of the PGRMC1 protein level from Figure 4**a**. Data are represented as mean ±SD. N=3 independent experiments. One-tailed Standard Student's t test was used to determine statistical significance. **b** Quantification of the RTN3 protein level from Figure 4**a**. Data are represented as mean ±SD. N=3 independent experiments. One-tailed Standard Student's t test was used to determine statistical significance. **c** Schematic of pre-proinsulin. Grey circles = signal peptide, blue circles = B-chain, green circles = C-peptide, pink circles = A chain, and white circles = prohormone convertase cleavage sites. Yellow variants represent proinsulin MIDY mutants tested in Figure 4**a**. Red circles refer to the MIDY mutants tested. **d** Schematic of oxidized proinsulin in the ER that exits this compartment to reach the secretory granule (where the C-peptide is normally excised) prior to secretion. Source data are provided as a Source Data file.

Figure S4 PGRMC1 is required for disposal of A16P. (Related to Figure 5)

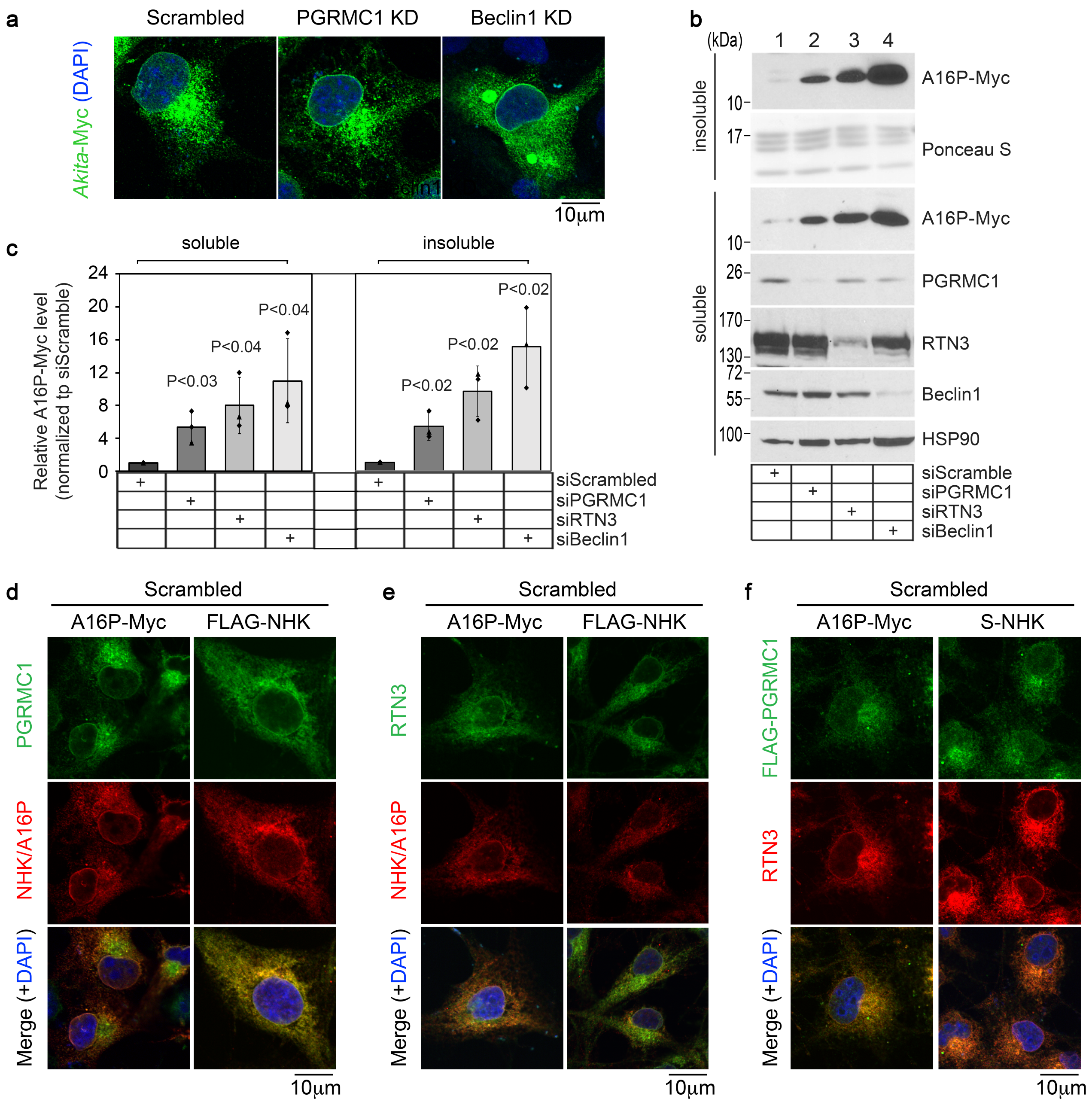

Figure S4. PGRMC1 is required for disposal of A16P. (Related to Figure 5). a As in Figure 5**b**, except cells were expressing *Akita*-Myc. N=3 independent experiments. **b** As in Figure 4**a**, except cells were transfected with the indicated siRNAs. N=3 independent experiments. **c** Quantification of soluble and insoluble A16P-Myc relative to the scrambled siRNA condition. Data are representative of the mean ±SD. N=3 independent experiments. One-tailed Standard Student's t test was used to determine statistical significance. **d-f** As in Figure 5**d-f**, but cells were transfected with the scrambled siRNA. N=3 independent experiments. Source data are provided as a Source Data file.

Figure S5 PGRMC1 targets small cargoes. (Related to Figure 6)

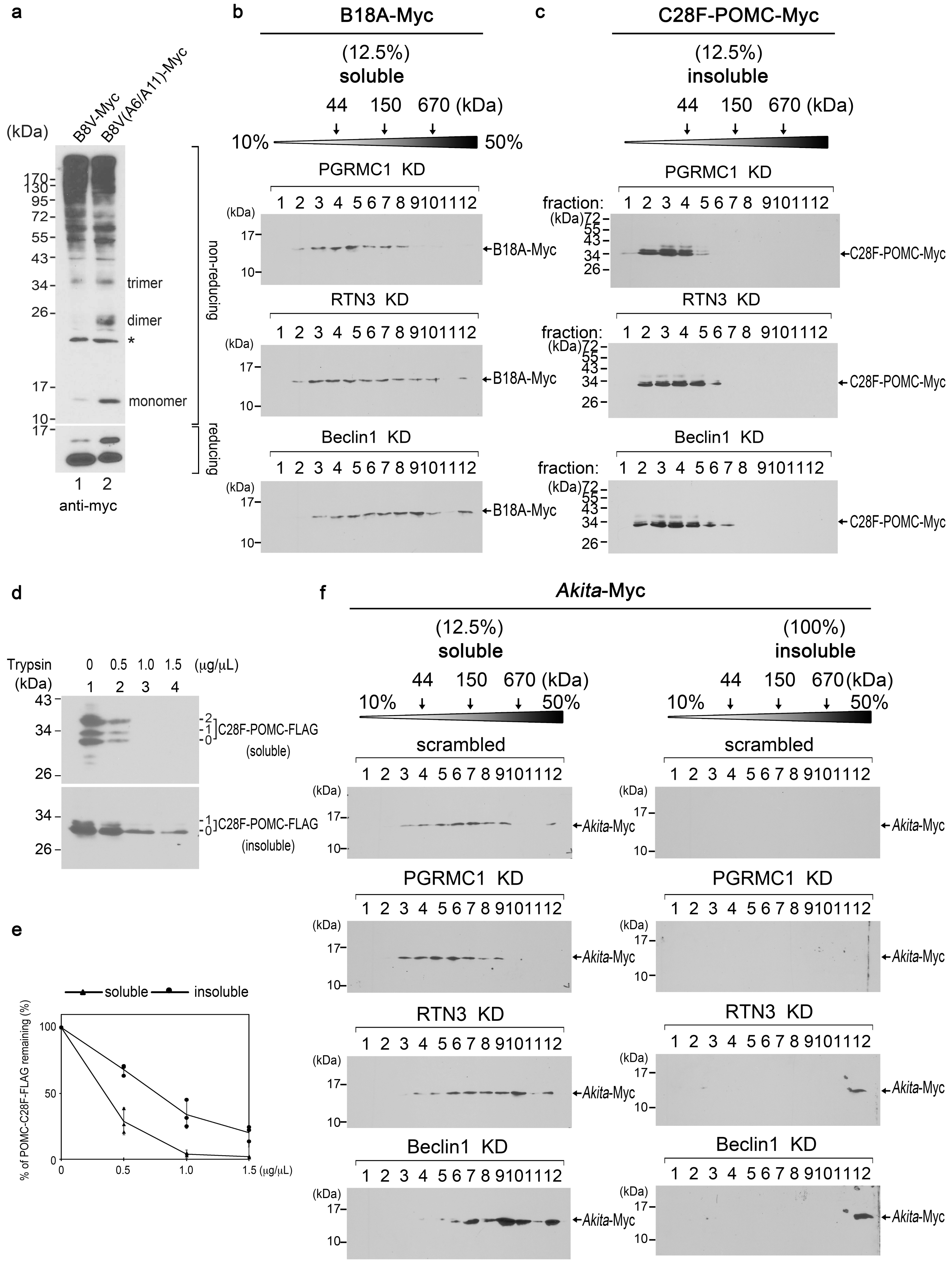

Figure S5. PGRMC1 targets small cargoes. (Related to Figure 6). a Extracts derived from HEK 293T cells expressing B8V-Myc or B8V(A6/A11)-Myc were subjected to non-reducing and reducing SDS-PAGE and immunoblotted with a Myc antibody. N=3 independent experiments. **b-c** Sucrose fractionation was performed as in Figure 6, except cells were transfected with the indicated mutant prohormone substrates. N=3 independent experiments. **d** HEK 293T cells expressing C28F-POMC-FLAG were lysed and separated into soluble and insoluble fractions as in 2**a**. Insoluble material was solubilized using 2% SDS, and the final SDS concentration was adjusted to .1% SDS. Extracts were treated with the indicated concentrations of trypsin and analyzed by SDS-PAGE and immunoblotting. N=3 independent experiments. **e** Quantification of remaining C28F-POMC-FLAG signal from S5D, normalized to lane 1 (no trypsin). Data are representative of the mean ±SD. N=3 independent experiments. **f** Sucrose fraction was performed as in Figure 6 using HEK 239T cells transfected *Akita*-myc. N=3 independent experiments. Source data are provided as a Source Data file.

Figure S6 Loss of PGRMC1-mediated ER-phagy increases proinsulin trafficking without causing ER stress. (Related to Figure 7)

a

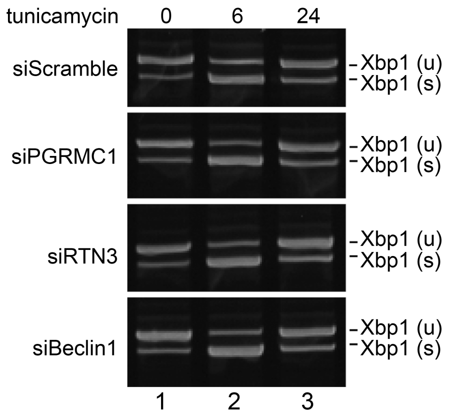

b

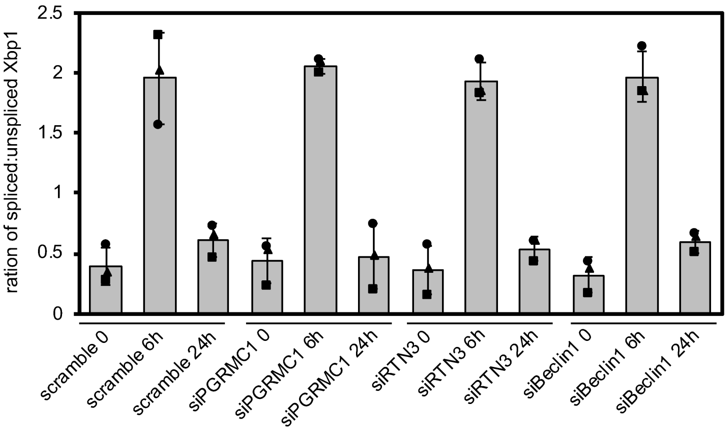

c

mouse islets

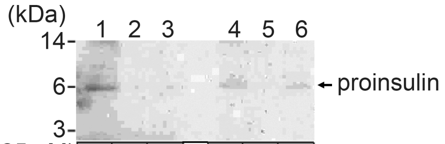

AG-205 (25 uM)					+	+	+
Cells (C) or Media (M)	C	C	M		C	C	M
Chase time (hr)	0	3	3		0	3	3

Figure S6. Loss of PGRMC1-mediated ER-phagy increases proinsulin trafficking without causing ER stress. (Related to Figure 7). a HEK 293T cells treated with the indicated siRNA s were exposed to tunicamycin (5 ug/mL) for 0, 6, or 24 hours. The splicing of Xbp1 was analyzed by PCR using primers flanking the IRE1 splice site as described previously. N=3 independent experiments. **b** Quantification of the ratio of spliced:unspliced Xbp1 from **a**. Data are representative of the mean ±SD. N=3 independent experiments. **c** B6 mouse islets were pulse-labeled with ^{35}S-labeled amino acids for 15 min in DMSO or 25 mM AG-205 containing media lacking Cys and Met, and chased for 3 h in DMSO or 25 mM AG-205 containing complete growth media. Islets were lysed immediately after pulse (0 chase time) or after chase. Media was collected at chase time. Islet lysates and media were immunoprecipitated with rabbit anti-mouse proinsulin-1 and -2 antibodies and protein A agarose overnight at 4°C. Immunoprecipitates were analyzed by nonreducing NuPAGE SDS-PAGE. Gels were fixed and dried, followed by phosphorimaging. An arrow shows the position of proinsulin. N=2 independent experiments. Source data are provided as a Source Data file.

Table S1. A list of all mass spectrometry results. (Related to Figure 1).
A list of all mass spectrometry hits for each of RTN3C, Sec61β, and RTN4A.

Mass spectrometry analysis of potential RTN3C binding partners

Reference	Gene Symbol	Unique peptides	Total peptides	Sum Intensity	Intensity%
sp|P53621|COPA_HUMAN	COPA	17	25	9.90E+06	1.21
sp|P10809|CH60_HUMAN	HSPD1	15	25	1.50E+07	1.81
sp|P68371|TBB4B_HUMAN	TUBB4B	13	21	1.40E+07	1.71
sp|Q9BQE3|TBA1C_HUMAN	TUBA1C	11	24	2.80E+07	3.49
sp|P06733|ENOA_HUMAN	ENO1	10	18	1.10E+07	1.36
sp|P14618|KPYM_HUMAN	PKM	10	17	7.50E+06	0.92
sp|P35606|COPB2_HUMAN	COPB2	10	17	6.30E+06	0.77
sp|P0DMV9|HS71B_HUMAN	HSPA1B	9	17	1.30E+07	1.57
sp|P07900|HS90A_HUMAN	HSP90AA1	9	14	6.90E+06	0.84
sp|P13639|EF2_HUMAN	EEF2	9	14	5.40E+06	0.66
sp|P06576|ATPB_HUMAN	ATP5B	9	13	4.10E+06	0.51
sp|P11021|GRP78_HUMAN	HSPA5	9	11	5.30E+06	0.65
sp|P04075|ALDOA_HUMAN	ALDOA	8	12	5.00E+06	0.61
sp|Q9Y678|COPG1_HUMAN	COPG1	8	9	2.50E+06	0.30
sp|P34931|HS71L_HUMAN	HSPA1L	7	14	1.40E+07	1.70
sp|P38646|GRP75_HUMAN	HSPA9	7	14	5.10E+06	0.63
sp|P62736|ACTA_HUMAN	ACTA2	7	13	1.40E+07	1.69
sp|P11142|HSP7C_HUMAN	HSPA8	7	12	8.90E+06	1.09
sp|P68104|EF1A1_HUMAN	EEF1A1	7	12	9.30E+06	1.14
sp|P25705|ATPA_HUMAN	ATP5A1	7	9	3.60E+06	0.44
sp|P19338|NUCL_HUMAN	NCL	7	7	2.50E+06	0.31
IGH1M_MOUSE	Ighg1	6	21	1.50E+08	18.23
sp|P12277|KCRB_HUMAN	CKB	6	10	4.10E+06	0.51
sp|P00558|PGK1_HUMAN	PGK1	6	9	2.20E+06	0.28
sp|P00338|LDHA_HUMAN	LDHA	6	6	3.50E+06	0.43
sp|P63261|ACTG_HUMAN	ACTG1	5	14	2.00E+07	2.40
sp|P07195|LDHB_HUMAN	LDHB	5	11	8.30E+06	1.01
sp|P04406|G3P_HUMAN	GAPDH	5	10	7.90E+06	0.97
sp|P06748|NPM_HUMAN	NPM1	5	9	5.90E+06	0.72
sp|P48444|COPD_HUMAN	ARCN1	5	8	2.60E+06	0.32
sp|P62258|1433E_HUMAN	YWHAE	5	8	2.80E+06	0.35
sp|P53618|COPB_HUMAN	COPB1	5	8	3.80E+06	0.46
sp|P61604|CH10_HUMAN	HSPE1	5	8	6.20E+06	0.76
sp|P23396|RS3_HUMAN	RPS3	5	6	2.20E+06	0.27
sp|P14625|ENPL_HUMAN	HSP90B1	5	6	3.00E+06	0.36
sp|Q06830|PRDX1_HUMAN	PRDX1	5	6	2.90E+06	0.36
sp|P32119|PRDX2_HUMAN	PRDX2	5	6	2.80E+06	0.34
sp|Q07021|C1QBP_HUMAN	C1QBP	4	9	3.70E+06	0.45
sp|P05387|RLA2_HUMAN	RPLP2	4	8	2.70E+06	0.33
sp|P07437|TBB5_HUMAN	TUBB	4	8	5.90E+06	0.73
sp|P40926|MDHM_HUMAN	MDH2	4	8	2.90E+06	0.35
sp|P54652|HSP72_HUMAN	HSPA2	4	7	4.20E+06	0.52
sp|P26641|EF1G_HUMAN	EEF1G	4	6	2.00E+06	0.25

sp|P51572|BAP31_HUMAN	BCAP31	4	5	1.90E+06	0.23
sp|P61204|ARF3_HUMAN	ARF3	4	4	1.50E+06	0.19
sp|Q02878|RL6_HUMAN	RPL6	4	4	2.40E+06	0.29
IGKC_MOUSE		3	12	7.30E+07	8.93
sp|P08238|HS90B_HUMAN	HSP90AB1	3	9	6.10E+06	0.75
sp|Q01105|SET_HUMAN	SET	3	6	1.40E+06	0.17
sp|P23528|COF1_HUMAN	CFL1	3	5	1.20E+06	0.14
sp|Q07020|RL18_HUMAN	RPL18	3	5	1.60E+06	0.20
sp|Q58FF8|H90B2_HUMAN	HSP90AB2I	3	5	7.10E+06	0.87
sp|P62241|RS8_HUMAN	RPS8	3	5	2.10E+06	0.26
sp|P60842|IF4A1_HUMAN	EIF4A1	3	5	1.40E+06	0.17
sp|Q58FF7|H90B3_HUMAN	HSP90AB3I	3	5	5.40E+06	0.66
sp|Q58FF6|H90B4_HUMAN	HSP90AB4I	3	5	4.70E+06	0.57
sp|P50990|TCPQ_HUMAN	CCT8	3	4	1.10E+06	0.14
sp|P62826|RAN_HUMAN	RAN	3	4	1.70E+06	0.21
sp|P15880|RS2_HUMAN	RPS2	3	4	1.30E+06	0.16
sp|P12273|PIP_HUMAN	PIP	3	4	9.90E+05	0.12
sp|P46781|RS9_HUMAN	RPS9	3	4	1.20E+06	0.15
sp|P62917|RL8_HUMAN	RPL8	3	3	9.20E+05	0.11
sp|P55072|TERA_HUMAN	VCP	3	3	5.60E+05	0.07
sp|P63244|RACK1_HUMAN	RACK1	3	3	1.20E+06	0.14
sp|P31948|STIP1_HUMAN	STIP1	3	3	7.10E+05	0.09
sp|P61978|HNRPK_HUMAN	HNRNPK	3	3	8.10E+05	0.10
sp|Q9NQC3|RTN4_HUMAN	RTN4	2	5	4.30E+06	0.53
KV2A7_MOUSE		2	5	1.50E+08	18.32
sp|P13929|ENOB_HUMAN	ENO3	2	5	4.00E+06	0.49
sp|P08865|RSSA_HUMAN	RPSA	2	4	1.00E+06	0.12
sp|O14579|COPE_HUMAN	COPE	2	4	1.90E+06	0.24
sp|Q13765|NACA_HUMAN	NACA	2	4	8.90E+05	0.11
sp|Q14568|HS902_HUMAN	HSP90AA2I	2	4	1.50E+06	0.18
sp|P22314|UBA1_HUMAN	UBA1	2	4	4.00E+05	0.05
sp|P62851|RS25_HUMAN	RPS25	2	4	2.30E+06	0.28
sp|Q15084|PDIA6_HUMAN	PDIA6	2	3	7.00E+05	0.09
sp|P62913|RL11_HUMAN	RPL11	2	3	7.70E+05	0.09
sp|P28066|PSA5_HUMAN	PSMA5	2	3	7.20E+05	0.09
sp|P63104|1433Z_HUMAN	YWHAZ	2	3	9.20E+05	0.11
sp|P36578|RL4_HUMAN	RPL4	2	3	1.00E+06	0.13
sp|P83731|RL24_HUMAN	RPL24	2	3	7.90E+05	0.10
sp|P19474|RO52_HUMAN	TRIM21	2	3	1.50E+06	0.19
sp|P42766|RL35_HUMAN	RPL35	2	3	1.20E+06	0.15
sp|Q92945|FUBP2_HUMAN	KHSRP	2	3	5.10E+05	0.06
sp|P62277|RS13_HUMAN	RPS13	2	3	7.90E+05	0.10
sp|Q6NVV1|R13P3_HUMAN	RPL13AP3	2	3	9.50E+05	0.12
sp|O43175|SERA_HUMAN	PHGDH	2	3	1.10E+06	0.13
sp|Q9HDC9|APMAP_HUMAN	APMAP	2	3	9.70E+05	0.12
sp|O95197|RTN3_HUMAN	RTN3	2	2	2.10E+06	0.26
sp|P30041|PRDX6_HUMAN	PRDX6	2	2	6.60E+05	0.08
sp|P26373|RL13_HUMAN	RPL13	2	2	5.60E+05	0.07

sp|P62820|RAB1A_HUMAN	RAB1A	2	2	3.80E+05	0.05
sp|P30048|PRDX3_HUMAN	PRDX3	2	2	5.30E+05	0.07
sp|P62701|RS4X_HUMAN	RPS4X	2	2	4.40E+05	0.05
sp|P84098|RL19_HUMAN	RPL19	2	2	5.70E+05	0.07
sp|P37802|TAGL2_HUMAN	TAGLN2	2	2	4.90E+05	0.06
sp|O00264|PGRC1_HUMAN	PGRMC1	2	2	5.80E+05	0.07
sp|P62249|RS16_HUMAN	RPS16	2	2	7.30E+05	0.09
sp|P22392|NDKB_HUMAN	NME2	2	2	6.90E+05	0.08
sp|Q04837|SSBP_HUMAN	SSBP1	2	2	3.90E+05	0.05
sp|P07737|PROF1_HUMAN	PFN1	2	2	8.50E+05	0.10
sp|P62987|RL40_HUMAN	UBA52	1	4	1.30E+07	1.63
sp|P51553|IDH3G_HUMAN	IDH3G	1	4	2.60E+07	3.16
sp|P50914|RL14_HUMAN	RPL14	1	3	1.10E+06	0.13
sp|Q9BVA1|TBB2B_HUMAN	TUBB2B	1	3	5.10E+06	0.62
sp|P62829|RL23_HUMAN	RPL23	1	3	1.10E+06	0.13
sp|P55209|NP1L1_HUMAN	NAP1L1	1	3	3.60E+05	0.04
sp|P62753|RS6_HUMAN	RPS6	1	3	1.60E+06	0.20
sp|P18669|PGAM1_HUMAN	PGAM1	1	3	5.30E+05	0.06
sp|O15173|PGRC2_HUMAN	PGRMC2	1	2	3.00E+05	0.04
sp|O75396|SC22B_HUMAN	SEC22B	1	2	4.00E+05	0.05
sp|P62424|RL7A_HUMAN	RPL7A	1	2	9.40E+05	0.12
sp|Q9NQ39|RS10L_HUMAN	RPS10P5	1	2	7.20E+05	0.09
sp|P00441|SODC_HUMAN	SOD1	1	2	6.50E+05	0.08
sp|P62857|RS28_HUMAN	RPS28	1	2	6.50E+05	0.08
sp|P61626|LYSC_HUMAN	LYZ	1	2	6.70E+05	0.08
sp|P07355|ANXA2_HUMAN	ANXA2	1	2	3.60E+05	0.04
sp|Q15517|CDSN_HUMAN	CDSN	1	2	8.10E+05	0.10
sp|P29692|EF1D_HUMAN	EEF1D	1	2	5.30E+05	0.06
sp|Q14240|IF4A2_HUMAN	EIF4A2	1	2	6.10E+05	0.07
sp|P48643|TCPE_HUMAN	CCT5	1	2	1.40E+05	0.02
sp|O14744|ANM5_HUMAN	PRMT5	1	2	7.70E+05	0.10
sp|P08559|ODPA_HUMAN	PDHA1	1	2	3.20E+05	0.04
sp|P25311|ZA2G_HUMAN	AZGP1	1	2	9.30E+05	0.11
sp|Q8NC51|PAIRB_HUMAN	SERBP1	1	2	2.40E+05	0.03
sp|P00387|NB5R3_HUMAN	CYB5R3	1	2	5.90E+05	0.07
sp|Q14103|HNRPD_HUMAN	HNRNPD	1	2	6.00E+05	0.07
sp|P62750|RL23A_HUMAN	RPL23A	1	2	1.00E+06	0.13
sp|P61923|COPZ1_HUMAN	COPZ1	1	2	5.00E+05	0.06
sp|P62937|PPIA_HUMAN	PPIA	1	2	1.50E+06	0.19
sp|P00492|HPRT_HUMAN	HPRT1	1	2	4.00E+05	0.05
sp|P07951|TPM2_HUMAN	TPM2	1	2	6.90E+05	0.08
sp|P24534|EF1B_HUMAN	EEF1B2	1	1	3.30E+05	0.04
sp|P16989|YBOX3_HUMAN	YBX3	1	1	1.60E+05	0.02
sp|P00505|AATM_HUMAN	GOT2	1	1	1.80E+05	0.02
sp|P67809|YBOX1_HUMAN	YBX1	1	1	2.30E+05	0.03
sp|P50991|TCPD_HUMAN	CCT4	1	1	2.50E+05	0.03
sp|P62854|RS26_HUMAN	RPS26	1	1	1.70E+05	0.02
sp|Q13283|G3BP1_HUMAN	G3BP1	1	1	1.90E+05	0.02

tr|H7BZJ3|H7BZJ3_HUMAN	PDIA3	1	1	2.00E+05	0.02
sp|P18621|RL17_HUMAN	RPL17	1	1	3.00E+05	0.04
sp|P35268|RL22_HUMAN	RPL22	1	1	4.00E+05	0.05
sp|P10599|THIO_HUMAN	TXN	1	1	2.90E+05	0.04
sp|P07237|PDIA1_HUMAN	P4HB	1	1	1.00E+05	0.01
sp|P23526|SAHH_HUMAN	AHCY	1	1	3.40E+05	0.04
sp|P28072|PSB6_HUMAN	PSMB6	1	1	2.20E+05	0.03
sp|P46776|RL27A_HUMAN	RPL27A	1	1	7.20E+05	0.09
sp|P50502|F10A1_HUMAN	ST13	1	1	2.30E+05	0.03
sp|P27348|1433T_HUMAN	YWHAQ	1	1	7.20E+04	0.01
sp|Q9BVC6|TM109_HUMAN	TMEM109	1	1	3.30E+05	0.04
sp|Q9Y3U8|RL36_HUMAN	RPL36	1	1	1.80E+05	0.02
sp|P39687|AN32A_HUMAN	ANP32A	1	1	2.10E+05	0.03
sp|Q15365|PCBP1_HUMAN	PCBP1	1	1	2.00E+05	0.02
sp|P78371|TCPB_HUMAN	CCT2	1	1	1.30E+05	0.02
sp|P22626|ROA2_HUMAN	HNRNPA2B	1	1	2.90E+05	0.04
sp|P15531|NDKA_HUMAN	NME1	1	1	2.80E+05	0.03
sp|P08670|VIME_HUMAN	VIM	1	1	2.80E+05	0.03
sp|Q04760|LGUL_HUMAN	GLO1	1	1	1.60E+05	0.02
sp|O00425|IF2B3_HUMAN	IGF2BP3	1	1	1.70E+05	0.02

Mass spectrometry analysis of potential Sec61β binding partners

Reference	Gene Symbol	Unique	Total	Sum Intensity	Intensity%
sp|P98175|RBM10_HUMAN	RBM10	18	28	4.80E+07	4.22
sp|P53621|COPA_HUMAN	COPA	18	23	1.70E+07	1.52
sp|P02788|TRFL_HUMAN	LTF	16	30	2.80E+07	2.43
sp|P35606|COPB2_HUMAN	COPB2	16	22	9.00E+06	0.78
sp|Q8TDL5|BPIB1_HUMAN	BPIFB1	13	17	1.30E+07	1.17
sp|P23396|RS3_HUMAN	RPS3	12	21	3.10E+07	2.67
sp|P68371|TBB4B_HUMAN	TUBB4B	12	19	9.40E+06	0.82
sp|P52272|HNRPM_HUMAN	HNRNPM	12	15	4.50E+06	0.39
sp|P10809|CH60_HUMAN	HSPD1	12	14	8.40E+06	0.74
sp|P53618|COPB_HUMAN	COPB1	11	17	5.30E+06	0.47
sp|O75533|SF3B1_HUMAN	SF3B1	11	12	3.40E+06	0.30
sp|O15042|SR140_HUMAN	U2SURP	10	12	6.20E+06	0.54
sp|Q9Y678|COPG1_HUMAN	COPG1	10	11	9.30E+06	0.81
sp|Q9BQE3|TBA1C_HUMAN	TUBA1C	9	18	2.00E+07	1.74
sp|P25311|ZA2G_HUMAN	AZGP1	9	14	8.00E+06	0.70
sp|Q9NZI8|IF2B1_HUMAN	IGF2BP1	9	13	5.20E+06	0.45
sp|P11021|GRP78_HUMAN	HSPA5	9	13	5.10E+06	0.45
sp|P06733|ENOA_HUMAN	ENO1	9	11	4.80E+06	0.42
sp|Q15393|SF3B3_HUMAN	SF3B3	9	11	2.70E+06	0.23
sp|Q9UHI6|DDX20_HUMAN	DDX20	9	10	6.70E+06	0.59
sp|P62269|RS18_HUMAN	RPS18	9	10	7.30E+06	0.64
sp|P11142|HSP7C_HUMAN	HSPA8	8	12	3.20E+06	0.28
sp|P07900|HS90A_HUMAN	HSP90AA1	8	11	3.60E+06	0.31
sp|P19338|NUCL_HUMAN	NCL	8	11	5.70E+06	0.50
sp|P68104|EF1A1_HUMAN	EEF1A1	8	10	7.50E+06	0.65
sp|P57678|GEMI4_HUMAN	GEMIN4	8	10	2.80E+06	0.24
sp|P14618|KPYM_HUMAN	PKM	8	10	3.10E+06	0.27
sp|P25705|ATPA_HUMAN	ATP5A1	8	10	5.10E+06	0.45
sp|P13639|EF2_HUMAN	EEF2	8	10	2.30E+06	0.20
sp|Q15233|NONO_HUMAN	NONO	8	10	5.10E+06	0.45
sp|P48444|COPD_HUMAN	ARCN1	8	9	3.40E+06	0.29
sp|P46781|RS9_HUMAN	RPS9	8	8	6.30E+06	0.55
sp|P04843|RPN1_HUMAN	RPN1	7	11	4.00E+06	0.35
sp|P08865|RSSA_HUMAN	RPSA	7	10	7.60E+06	0.66
sp|P11940|PABP1_HUMAN	PABPC1	7	9	3.40E+06	0.30
sp|P06576|ATPB_HUMAN	ATP5B	7	7	1.60E+06	0.14
sp|Q15208|STK38_HUMAN	STK38	6	11	4.40E+06	0.38
sp|P63261|ACTG_HUMAN	ACTG1	6	10	7.40E+06	0.65
sp|P04083|ANXA1_HUMAN	ANXA1	6	10	3.30E+06	0.28
sp|P62701|RS4X_HUMAN	RPS4X	6	10	8.40E+06	0.74
sp|Q8NHW5|RLA0L_HUMAN	RPLP0P6	6	9	4.10E+06	0.36
sp|P0DMV9|HS71B_HUMAN	HSPA1B	6	8	3.10E+06	0.27
sp|Q96DA0|ZG16B_HUMAN	ZG16B	6	8	7.20E+06	0.63
sp|Q96I25|SPF45_HUMAN	RBM17	6	8	2.20E+06	0.20

sp|P15880|RS2_HUMAN	RPS2	6	8	7.70E+06	0.67
sp|P07195|LDHB_HUMAN	LDHB	6	8	2.70E+06	0.23
sp|P06748|NPM_HUMAN	NPM1	6	7	1.50E+07	1.28
sp|P23246|SFPQ_HUMAN	SFPQ	6	7	2.50E+06	0.22
sp|O14744|ANM5_HUMAN	PRMT5	6	7	2.70E+06	0.23
sp|O43143|DHX15_HUMAN	DHX15	6	7	2.70E+06	0.23
sp|Q13435|SF3B2_HUMAN	SF3B2	6	6	2.40E+06	0.21
IGH1M_MOUSE	Ighg1	5	14	8.20E+07	7.16
sp|P04406|G3P_HUMAN	GAPDH	5	9	2.20E+07	1.91
sp|Q9NP55|BPIA1_HUMAN	BPIFA1	5	9	7.10E+06	0.62
sp|P12273|PIP_HUMAN	PIP	5	7	6.10E+06	0.53
sp|P61247|RS3A_HUMAN	RPS3A	5	7	1.30E+07	1.10
sp|P38646|GRP75_HUMAN	HSPA9	5	7	1.60E+06	0.14
sp|P62258|1433E_HUMAN	YWHAE	5	6	3.90E+06	0.34
sp|P36578|RL4_HUMAN	RPL4	5	6	2.00E+06	0.18
sp|P26373|RL13_HUMAN	RPL13	5	6	2.80E+06	0.25
sp|P62917|RL8_HUMAN	RPL8	5	5	3.20E+06	0.28
sp|P54652|HSP72_HUMAN	HSPA2	5	5	2.30E+06	0.20
sp|P62249|RS16_HUMAN	RPS16	5	5	4.90E+06	0.43
sp|Q06830|PRDX1_HUMAN	PRDX1	5	5	1.80E+06	0.16
sp|Q9Y520|PRC2C_HUMAN	PRRC2C	5	5	8.50E+05	0.07
sp|P62280|RS11_HUMAN	RPS11	5	5	4.40E+06	0.39
sp|P62277|RS13_HUMAN	RPS13	5	5	3.90E+06	0.34
sp|Q9UQ35|SRRM2_HUMAN	SRRM2	5	5	9.10E+05	0.08
sp|P01876|IGHA1_HUMAN	IGHA1	4	9	6.80E+07	5.90
sp|P39023|RL3_HUMAN	RPL3	4	8	1.80E+06	0.16
sp|P62736|ACTA_HUMAN	ACTA2	4	8	6.10E+06	0.53
IGKC_MOUSE		4	7	4.30E+07	3.74
sp|Q92841|DDX17_HUMAN	DDX17	4	7	2.80E+06	0.25
sp|P62241|RS8_HUMAN	RPS8	4	5	4.80E+06	0.42
sp|P62424|RL7A_HUMAN	RPL7A	4	5	2.60E+06	0.23
sp|Q14152|EIF3A_HUMAN	EIF3A	4	5	1.20E+06	0.10
sp|Q9Y262|EIF3L_HUMAN	EIF3L	4	5	1.40E+06	0.12
sp|P55884|EIF3B_HUMAN	EIF3B	4	5	1.60E+06	0.14
sp|Q06787|FMR1_HUMAN	FMR1	4	5	1.90E+06	0.17
sp|P61626|LYSC_HUMAN	LYZ	4	5	2.60E+07	2.27
sp|P09211|GSTP1_HUMAN	GSTP1	4	5	6.20E+05	0.05
sp|P39019|RS19_HUMAN	RPS19	4	5	5.50E+06	0.48
sp|P12277|KCRB_HUMAN	CKB	4	4	7.30E+05	0.06
sp|Q9HC84|MUC5B_HUMAN	MUC5B	4	4	7.90E+05	0.07
sp|P61204|ARF3_HUMAN	ARF3	4	4	8.00E+05	0.07
sp|P04040|CATA_HUMAN	CAT	4	4	6.00E+05	0.05
sp|P04075|ALDOA_HUMAN	ALDOA	4	4	1.10E+06	0.10
sp|Q9UGM3|DMBT1_HUMAN	DMBT1	4	4	6.00E+06	0.53
sp|Q7KZF4|SND1_HUMAN	SND1	4	4	8.90E+05	0.08
sp|P62263|RS14_HUMAN	RPS14	4	4	4.40E+06	0.38
sp|P0DOX2|IGA2_HUMAN		4	4	4.70E+07	4.06
sp|Q02878|RL6_HUMAN	RPL6	4	4	2.60E+06	0.23

sp|Q9BRS2|RIOK1_HUMAN	RIOK1	4	4	8.00E+05	0.07
sp|P00338|LDHA_HUMAN	LDHA	4	4	1.30E+06	0.11
sp|P62266|RS23_HUMAN	RPS23	4	4	2.30E+06	0.20
sp|Q9NTJ5|SAC1_HUMAN	SACM1L	4	4	6.10E+05	0.05
sp|P39656|OST48_HUMAN	DDOST	4	4	8.90E+05	0.08
sp|P60468|SC61B_HUMAN	SEC61B	4	4	4.30E+06	0.37
sp|P22626|ROA2_HUMAN	HNRNPA2B1	4	4	1.10E+06	0.10
sp|Q9NVI7|ATD3A_HUMAN	ATAD3A	4	4	7.00E+05	0.06
sp|P14625|ENPL_HUMAN	HSP90B1	4	4	9.00E+05	0.08
sp|P59827|BPIB4_HUMAN	BPIFB4	4	4	6.30E+05	0.06
sp|P07437|TBB5_HUMAN	TUBB	3	8	4.50E+06	0.40
sp|P05388|RLA0_HUMAN	RPLP0	3	6	1.00E+06	0.09
sp|P05387|RLA2_HUMAN	RPLP2	3	6	3.20E+06	0.28
sp|Q07021|C1QBP_HUMAN	C1QBP	3	6	1.80E+06	0.15
sp|P62847|RS24_HUMAN	RPS24	3	6	2.30E+06	0.20
sp|P62244|RS15A_HUMAN	RPS15A	3	5	3.10E+06	0.27
sp|P01619|KV320_HUMAN	IGKV3-20	3	5	2.70E+06	0.24
sp|P62753|RS6_HUMAN	RPS6	3	5	2.90E+06	0.25
sp|P33778|H2B1B_HUMAN	HIST1H2BB	3	5	2.00E+06	0.18
sp|P62805|H4_HUMAN	HIST1H4A	3	5	3.00E+06	0.26
sp|P62987|RL40_HUMAN	UBA52	3	4	1.30E+07	1.12
sp|P0DOX7|IGK_HUMAN		3	4	3.10E+06	0.27
sp|Q00839|HNRPU_HUMAN	HNRNPU	3	4	5.70E+05	0.05
sp|P51571|SSRD_HUMAN	SSR4	3	4	9.70E+06	0.85
sp|P63244|RACK1_HUMAN	RACK1	3	4	2.00E+06	0.17
sp|P14678|RSMB_HUMAN	SNRPB	3	4	7.00E+06	0.61
sp|Q58FF8|H90B2_HUMAN	HSP90AB2P	3	4	2.30E+06	0.20
sp|P62937|PPIA_HUMAN	PPIA	3	4	2.00E+06	0.17
sp|P05164|PERM_HUMAN	MPO	3	4	5.10E+05	0.04
sp|Q9BQA1|MEP50_HUMAN	WDR77	3	3	8.10E+05	0.07
sp|P61923|COPZ1_HUMAN	COPZ1	3	3	6.90E+05	0.06
sp|P63104|1433Z_HUMAN	YWHAZ	3	3	4.20E+05	0.04
sp|P07737|PROF1_HUMAN	PFN1	3	3	1.00E+06	0.09
sp|P62857|RS28_HUMAN	RPS28	3	3	1.80E+06	0.15
sp|P00738|HPT_HUMAN	HP	3	3	8.10E+05	0.07
sp|O00303|EIF3F_HUMAN	EIF3F	3	3	6.00E+05	0.05
sp|P61978|HNRPK_HUMAN	HNRNPK	3	3	6.30E+05	0.06
sp|P62851|RS25_HUMAN	RPS25	3	3	3.80E+06	0.34
sp|Q04837|SSBP_HUMAN	SSBP1	3	3	4.30E+05	0.04
sp|O43175|SERA_HUMAN	PHGDH	3	3	4.60E+05	0.04
sp|Q58FF7|H90B3_HUMAN	HSP90AB3P	3	3	1.20E+06	0.11
sp|Q9NR31|SAR1A_HUMAN	SAR1A	3	3	1.40E+06	0.12
sp|Q07955|SRSF1_HUMAN	SRSF1	3	3	7.10E+05	0.06
sp|P09874|PARP1_HUMAN	PARP1	3	3	3.60E+05	0.03
sp|P18124|RL7_HUMAN	RPL7	3	3	1.30E+06	0.11
sp|Q08211|DHX9_HUMAN	DHX9	3	3	5.10E+05	0.04
sp|P34931|HS71L_HUMAN	HSPA1L	3	3	1.50E+06	0.13
sp|P50990|TCPQ_HUMAN	CCT8	3	3	2.80E+05	0.02

sp|Q8TEQ6|GEMI5_HUMAN	GEMIN5	3	3	5.30E+05	0.05
sp|P40926|MDHM_HUMAN	MDH2	3	3	4.40E+05	0.04
sp|Q92499|DDX1_HUMAN	DDX1	3	3	3.90E+05	0.03
sp|Q14444|CAPR1_HUMAN	CAPRIN1	3	3	6.20E+05	0.05
sp|O15371|EIF3D_HUMAN	EIF3D	3	3	4.80E+05	0.04
sp|P0CG04|IGLC1_HUMAN	IGLC1	2	6	7.80E+06	0.68
sp|Q9BVA1|TBB2B_HUMAN	TUBB2B	2	5	4.80E+06	0.42
sp|P01857|IGHG1_HUMAN	IGHG1	2	4	9.60E+05	0.08
sp|P84098|RL19_HUMAN	RPL19	2	4	1.30E+06	0.11
sp|Q16629|SRSF7_HUMAN	SRSF7	2	4	7.90E+05	0.07
sp|P0DOY2|IGLC2_HUMAN	IGLC2	2	4	6.90E+06	0.61
sp|Q9NQ39|RS10L_HUMAN	RPS10P5	2	4	3.20E+06	0.28
sp|P22314|UBA1_HUMAN	UBA1	2	4	2.80E+05	0.02
sp|P08238|HS90B_HUMAN	HSP90AB1	2	3	5.60E+05	0.05
sp|P62081|RS7_HUMAN	RPS7	2	3	2.30E+06	0.20
sp|P61313|RL15_HUMAN	RPL15	2	3	8.10E+05	0.07
sp|Q9UN86|G3BP2_HUMAN	G3BP2	2	3	3.70E+05	0.03
sp|Q8IWX8|CHERP_HUMAN	CHERP	2	3	1.00E+06	0.09
KV2A7_MOUSE		2	3	7.80E+07	6.80
sp|P00558|PGK1_HUMAN	PGK1	2	3	5.20E+05	0.05
sp|P42677|RS27_HUMAN	RPS27	2	3	1.20E+06	0.11
sp|Q12905|ILF2_HUMAN	ILF2	2	3	3.50E+05	0.03
sp|P80188|NGAL_HUMAN	LCN2	2	3	3.70E+05	0.03
sp|Q9BWU0|NADAP_HUMAN	SLC4A1AP	2	3	3.20E+05	0.03
sp|Q6P5S2|LEG1H_HUMAN	LEG1	2	3	5.40E+05	0.05
sp|Q9UBF2|COPG2_HUMAN	COPG2	2	3	5.20E+05	0.05
sp|Q13247|SRSF6_HUMAN	SRSF6	2	3	5.00E+05	0.04
sp|Q96KK5|H2A1H_HUMAN	HIST1H2AH	2	3	5.10E+06	0.45
sp|P32969|RL9_HUMAN	RPL9	2	3	1.20E+06	0.10
sp|P46778|RL21_HUMAN	RPL21	2	2	2.30E+06	0.20
sp|P07355|ANXA2_HUMAN	ANXA2	2	2	1.30E+06	0.11
sp|O14579|COPE_HUMAN	COPE	2	2	6.40E+05	0.06
sp|O75396|SC22B_HUMAN	SEC22B	2	2	5.00E+05	0.04
sp|Q07020|RL18_HUMAN	RPL18	2	2	9.40E+05	0.08
sp|P63220|RS21_HUMAN	RPS21	2	2	1.10E+06	0.09
sp|P61604|CH10_HUMAN	HSPE1	2	2	6.50E+05	0.06
sp|Q9H3N1|TMX1_HUMAN	TMX1	2	2	5.90E+05	0.05
sp|P26599|PTBP1_HUMAN	PTBP1	2	2	5.90E+05	0.05
sp|Q8NC51|PAIRB_HUMAN	SERBP1	2	2	8.50E+05	0.07
sp|Q6NVV1|R13P3_HUMAN	RPL13AP3	2	2	1.70E+06	0.15
sp|P35268|RL22_HUMAN	RPL22	2	2	1.30E+06	0.11
sp|O75821|EIF3G_HUMAN	EIF3G	2	2	4.30E+05	0.04
sp|P23528|COF1_HUMAN	CFL1	2	2	2.80E+05	0.02
sp|P01766|HV313_HUMAN	IGHV3-13	2	2	2.50E+05	0.02
sp|Q93079|H2B1H_HUMAN	HIST1H2BH	2	2	5.10E+05	0.04
sp|P19474|RO52_HUMAN	TRIM21	2	2	3.20E+06	0.28
sp|P10599|THIO_HUMAN	TXN	2	2	1.80E+06	0.16
sp|P62318|SMD3_HUMAN	SNRPD3	2	2	7.40E+05	0.06

sp|P18621|RL17_HUMAN	RPL17	2	2	8.70E+05	0.08
sp|Q16637|SMN_HUMAN	SMN1	2	2	4.80E+05	0.04
sp|P62304|RUXE_HUMAN	SNRPE	2	2	4.90E+05	0.04
sp|O60506|HNRPQ_HUMAN	SYNCRIP	2	2	1.70E+05	0.02
sp|Q9Y3B4|SF3B6_HUMAN	SF3B6	2	2	4.60E+05	0.04
sp|Q15459|SF3A1_HUMAN	SF3A1	2	2	3.10E+05	0.03
sp|P49327|FAS_HUMAN	FASN	2	2	1.50E+05	0.01
sp|P62750|RL23A_HUMAN	RPL23A	2	2	1.00E+06	0.09
sp|P46777|RL5_HUMAN	RPL5	2	2	6.20E+05	0.05
sp|P07910|HNRPC_HUMAN	HNRNPC	2	2	4.30E+05	0.04
sp|Q8N3C0|ASCC3_HUMAN	ASCC3	2	2	1.30E+05	0.01
sp|Q12906|ILF3_HUMAN	ILF3	2	2	2.10E+05	0.02
sp|P46779|RL28_HUMAN	RPL28	2	2	9.20E+05	0.08
sp|P08621|RU17_HUMAN	SNRNP70	2	2	4.30E+05	0.04
sp|Q8NHQ1|CEP70_HUMAN	CEP70	2	2	1.50E+06	0.13
sp|O95969|SG1D2_HUMAN	SCGB1D2	2	2	1.90E+06	0.16
sp|P01591|IGJ_HUMAN	JCHAIN	2	2	2.20E+06	0.19
sp|P46783|RS10_HUMAN	RPS10	2	2	9.10E+05	0.08
sp|P05141|ADT2_HUMAN	SLC25A5	2	2	6.60E+05	0.06
sp|O43390|HNRPR_HUMAN	HNRNPR	2	2	5.40E+05	0.05
sp|Q00325|MPCP_HUMAN	SLC25A3	2	2	1.60E+06	0.14
sp|Q32P51|RA1L2_HUMAN	HNRNPA1L2	2	2	6.80E+05	0.06
sp|Q6PKG0|LARP1_HUMAN	LARP1	2	2	2.00E+05	0.02
sp|P01834|IGKC_HUMAN	IGKC	1	8	1.30E+07	1.13
sp|P08708|RS17_HUMAN	RPS17	1	6	1.00E+06	0.09
sp|Q16378|PROL4_HUMAN	PRR4	1	4	3.30E+06	0.29
sp|P02808|STAT_HUMAN	STATH	1	4	1.70E+06	0.15
sp|P62854|RS26_HUMAN	RPS26	1	4	2.90E+06	0.26
tr|L0R599|L0R599_HUMAN	CIRH1A	1	4	1.20E+07	1.01
tr|A0A0B4J2B5|A0A0B4J2B5_HUM	IGHV3OR16-9	1	3	1.50E+06	0.13
tr|Q9UL78|Q9UL78_HUMAN		1	3	8.10E+05	0.07
sp|Q15517|CDSN_HUMAN	CDSN	1	3	2.10E+06	0.18
sp|P62899|RL31_HUMAN	RPL31	1	3	1.10E+06	0.10
tr|B4DUE0|B4DUE0_HUMAN		1	3	6.10E+06	0.53
tr|A0A0X9TD47|A0A0X9TD47_HUMAN		1	2	3.40E+05	0.03
sp|P05386|RLA1_HUMAN	RPLP1	1	2	5.60E+05	0.05
tr|Q96SA9|Q96SA9_HUMAN		1	2	3.40E+05	0.03
sp|P16989|YBOX3_HUMAN	YBX3	1	2	2.10E+05	0.02
sp|Q9P035|HACD3_HUMAN	HACD3	1	2	6.50E+05	0.06
sp|Q9P003|CNIH4_HUMAN	CNIH4	1	2	3.20E+05	0.03
sp|P13929|ENOB_HUMAN	ENO3	1	2	9.30E+05	0.08
sp|P16615|AT2A2_HUMAN	ATP2A2	1	2	3.10E+05	0.03
sp|P80748|LV321_HUMAN	IGLV3-21	1	2	5.00E+05	0.04
sp|P50502|F10A1_HUMAN	ST13	1	2	2.00E+05	0.02
sp|O75223|GGCT_HUMAN	GGCT	1	2	3.70E+05	0.03
sp|Q02539|H11_HUMAN	HIST1H1A	1	2	3.10E+05	0.03
sp|Q8TB92|HMGC2_HUMAN	HMGCLL1	1	1	1.40E+06	0.12
tr|A0A193CHR0|A0A193CHR0_HUMAN		1	1	2.60E+05	0.02

tr|Q6IPH7|Q6IPH7_HUMAN	RPL14	1	1	1.90E+05	0.02
sp|P14314|GLU2B_HUMAN	PRKCSH	1	1	1.20E+07	1.05
sp|Q15428|SF3A2_HUMAN	SF3A2	1	1	1.60E+05	0.01
sp|O95292|VAPB_HUMAN	VAPB	1	1	2.50E+05	0.02
sp|P55209|NP1L1_HUMAN	NAP1L1	1	1	9.40E+04	0.01
sp|P52597|HNRPF_HUMAN	HNRNPF	1	1	1.10E+05	0.01
sp|P50914|RL14_HUMAN	RPL14	1	1	6.20E+05	0.05
sp|P00505|AATM_HUMAN	GOT2	1	1	1.20E+05	0.01
sp|Q9UNL2|SSRG_HUMAN	SSR3	1	1	1.20E+05	0.01
sp|O14893|GEMI2_HUMAN	GEMIN2	1	1	1.70E+05	0.01
sp|Q9BVK6|TMED9_HUMAN	TMED9	1	1	2.40E+05	0.02
sp|P62829|RL23_HUMAN	RPL23	1	1	4.90E+05	0.04
tr|B2R7Z6|B2R7Z6_HUMAN		1	1	9.60E+04	0.01
sp|Q9BVC6|TM109_HUMAN	TMEM109	1	1	5.80E+05	0.05
sp|O00425|IF2B3_HUMAN	IGF2BP3	1	1	4.20E+05	0.04
sp|A0A0B4J1X5|HV374_HUMAN	IGHV3-74	1	1	1.20E+05	0.01
sp|P20061|TCO1_HUMAN	TCN1	1	1	1.10E+05	0.01
tr|A0A0X9V9C4|A0A0X9V9C4_HUMAN		1	1	3.20E+05	0.03
sp|P61803|DAD1_HUMAN	DAD1	1	1	1.50E+05	0.01
sp|A8MWD9|RUXGL_HUMAN	SNRPGP15	1	1	2.10E+05	0.02
sp|P46776|RL27A_HUMAN	RPL27A	1	1	1.00E+06	0.09
sp|P67809|YBOX1_HUMAN	YBX1	1	1	7.50E+05	0.07
sp|Q9GZV4|IF5A2_HUMAN	EIF5A2	1	1	1.40E+05	0.01
sp|Q6ZVX7|FBX50_HUMAN	NCCRP1	1	1	2.60E+05	0.02
sp|P07996|TSP1_HUMAN	THBS1	1	1	1.20E+05	0.01
sp|P01037|CYTN_HUMAN	CST1	1	1	6.60E+05	0.06
sp|P55072|TERA_HUMAN	VCP	1	1	2.10E+05	0.02
sp|Q13283|G3BP1_HUMAN	G3BP1	1	1	1.30E+05	0.01
tr|A0A0A0MR66|A0A0A0MR66_HU	RBM10	1	1	5.20E+05	0.05
sp|P27482|CALL3_HUMAN	CALML3	1	1	9.60E+04	0.01
sp|P78527|PRKDC_HUMAN	PRKDC	1	1	1.00E+05	0.01
sp|O75477|ERLN1_HUMAN	ERLIN1	1	1	2.10E+05	0.02
sp|P35030|TRY3_HUMAN	PRSS3	1	1	1.10E+07	0.95
sp|Q13765|NACA_HUMAN	NACA	1	1	1.00E+05	0.01
sp|Q9BRL6|SRSF8_HUMAN	SRSF8	1	1	1.70E+05	0.01
sp|P54105|ICLN_HUMAN	CLNS1A	1	1	9.20E+04	0.01
sp|Q9Y3F4|STRAP_HUMAN	STRAP	1	1	2.40E+05	0.02
sp|Q9UBC9|SPRR3_HUMAN	SPRR3	1	1	1.10E+05	0.01
sp|Q8WVV4|POF1B_HUMAN	POF1B	1	1	1.00E+05	0.01
sp|P47813|IF1AX_HUMAN	EIF1AX	1	1	1.80E+05	0.02
sp|P29692|EF1D_HUMAN	EEF1D	1	1	1.40E+05	0.01
sp|P50395|GDIB_HUMAN	GDI2	1	1	8.20E+04	0.01
sp|P69849|NOMO3_HUMAN	NOMO3	1	1	4.00E+04	0.00
sp|P01034|CYTC_HUMAN	CST3	1	1	1.80E+05	0.02
sp|Q01105|SET_HUMAN	SET	1	1	2.00E+05	0.02
sp|Q6UWW0|LCN15_HUMAN	LCN15	1	1	7.00E+05	0.06
sp|P28074|PSB5_HUMAN	PSMB5	1	1	1.00E+05	0.01
sp|Q13296|SG2A2_HUMAN	SCGB2A2	1	1	1.60E+06	0.14

sp|P60900|PSA6_HUMAN	PSMA6	1	1	9.50E+04	0.01
sp|Q14240|IF4A2_HUMAN	EIF4A2	1	1	3.00E+05	0.03
sp|Q13867|BLMH_HUMAN	BLMH	1	1	1.50E+05	0.01
sp|O94905|ERLN2_HUMAN	ERLIN2	1	1	1.60E+05	0.01
sp|Q99613|EIF3C_HUMAN	EIF3C	1	1	3.80E+05	0.03
sp|P06396|GELS_HUMAN	GSN	1	1	8.90E+04	0.01
sp|P83731|RL24_HUMAN	RPL24	1	1	4.60E+05	0.04
sp|Q14568|HS902_HUMAN	HSP90AA2P	1	1	4.30E+05	0.04
sp|A6NIE6|RN3P2_HUMAN	RRN3P2	1	1	3.50E+06	0.31
sp|P32119|PRDX2_HUMAN	PRDX2	1	1	3.20E+05	0.03
sp|P49454|CENPF_HUMAN	CENPF	1	1	3.50E+05	0.03
sp|P62861|RS30_HUMAN	FAU	1	1	3.10E+05	0.03
sp|Q7L2E3|DHX30_HUMAN	DHX30	1	1	1.20E+05	0.01
sp|Q92945|FUBP2_HUMAN	KHSRP	1	1	1.10E+05	0.01
sp|Q9HDC9|APMAP_HUMAN	APMAP	1	1	1.10E+05	0.01
tr|Q8N355|Q8N355_HUMAN	IGL@	1	1	3.00E+06	0.26
sp|P61353|RL27_HUMAN	RPL27	1	1	6.40E+05	0.06
sp|O14983|AT2A1_HUMAN	ATP2A1	1	1	1.10E+05	0.01
sp|Q9UBQ5|EIF3K_HUMAN	EIF3K	1	1	1.60E+05	0.01
sp|Q02543|RL18A_HUMAN	RPL18A	1	1	2.60E+05	0.02
sp|O60449|LY75_HUMAN	LY75	1	1	1.70E+06	0.15
sp|P08670|VIME_HUMAN	VIM	1	1	7.00E+05	0.06
sp|Q9UI42|CBPA4_HUMAN	CPA4	1	1	1.40E+05	0.01
sp|P48634|PRC2A_HUMAN	PRRC2A	1	1	2.70E+05	0.02

Mass spectrometry analysis of potential RTN4A binding partners

Reference	Gene Symbol	Unique peptides	Total peptides	Sum Intensity	Intensity%
sp|Q9NQC3|RTN4_HUMAN	RTN4	43	97	2.20E+08	44.51
sp|Q15208|STK38_HUMAN	STK38	12	20	1.30E+07	2.73
sp|P14618|KPYM_HUMAN	PKM	10	17	5.60E+06	1.16
sp|P10809|CH60_HUMAN	HSPD1	10	16	7.30E+06	1.52
sp|P0DMV9|HS71B_HUMAN	HSPA1B	8	11	5.20E+06	1.08
sp|P04406|G3P_HUMAN	GAPDH	7	15	1.50E+07	3.04
sp|P68104|EF1A1_HUMAN	EEF1A1	7	13	7.80E+06	1.62
sp|P06733|ENOA_HUMAN	ENO1	7	10	5.10E+06	1.06
sp|P11142|HSP7C_HUMAN	HSPA8	7	10	4.20E+06	0.86
sp|Q9BQE3|TBA1C_HUMAN	TUBA1C	6	14	7.60E+06	1.57
sp|P11021|GRP78_HUMAN	HSPA5	6	10	5.00E+06	1.03
sp|P62736|ACTA_HUMAN	ACTA2	6	8	7.80E+06	1.62
sp|P34931|HS71L_HUMAN	HSPA1L	6	8	4.40E+06	0.92
sp|P13639|EF2_HUMAN	EEF2	6	7	1.70E+06	0.35
sp|P07900|HS90A_HUMAN	HSP90AA1	5	9	3.40E+06	0.69
sp|P04083|ANXA1_HUMAN	ANXA1	5	9	1.80E+06	0.37
sp|P68371|TBB4B_HUMAN	TUBB4B	5	9	5.20E+06	1.07
sp|P00338|LDHA_HUMAN	LDHA	5	5	1.80E+06	0.37
sp|P54652|HSP72_HUMAN	HSPA2	5	5	2.90E+06	0.60
IGH1M_MOUSE	Ighg1	4	14	3.20E+07	6.67
sp|P63261|ACTG_HUMAN	ACTG1	4	10	1.30E+07	2.73
IGKC_MOUSE		4	7	1.90E+07	3.88
sp|P25311|ZA2G_HUMAN	AZGP1	4	7	2.30E+06	0.48
sp|P07195|LDHB_HUMAN	LDHB	4	7	2.70E+06	0.56
sp|P38646|GRP75_HUMAN	HSPA9	4	6	2.20E+06	0.45
sp|P32119|PRDX2_HUMAN	PRDX2	4	6	5.00E+06	1.04
sp|P61204|ARF3_HUMAN	ARF3	4	5	2.20E+06	0.45
sp|P06576|ATPB_HUMAN	ATP5B	4	4	1.40E+06	0.29
sp|P07437|TBB5_HUMAN	TUBB	3	6	1.50E+06	0.31
sp|P62258|1433E_HUMAN	YWHAE	3	5	1.40E+06	0.29
sp|P62987|RL40_HUMAN	UBA52	3	4	7.50E+06	1.56
sp|P61604|CH10_HUMAN	HSPE1	3	4	1.90E+06	0.39
sp|Q06830|PRDX1_HUMAN	PRDX1	3	4	2.60E+06	0.54
sp|P12277|KCRB_HUMAN	CKB	3	4	1.10E+06	0.22
sp|Q8WVV4|POF1B_HUMAN	POF1B	3	4	1.10E+06	0.22
sp|Q58FF7|H90B3_HUMAN	HSP90AB3I	3	3	1.40E+06	0.30
sp|P04792|HSPB1_HUMAN	HSPB1	2	5	1.10E+06	0.23
sp|Q8N4C6|NIN_HUMAN	NIN	2	5	3.90E+06	0.80
sp|P08238|HS90B_HUMAN	HSP90AB1	2	4	1.80E+06	0.37
sp|P07355|ANXA2_HUMAN	ANXA2	2	4	2.70E+06	0.57
tr|A1A4E9|A1A4E9_HUMAN	KRT13	2	4	4.60E+06	0.95
sp|P07737|PROF1_HUMAN	PFN1	2	3	6.70E+05	0.14
sp|P08865|RSSA_HUMAN	RPSA	2	3	7.00E+05	0.14

sp|P12273|PIP_HUMAN	PIP	2	3	1.60E+06	0.33
sp|Q58FF6|H90B4_HUMAN	HSP90AB4I	2	3	1.40E+06	0.29
sp|P04075|ALDOA_HUMAN	ALDOA	2	3	1.10E+06	0.24
sp|O14744|ANM5_HUMAN	PRMT5	2	3	7.70E+05	0.16
sp|Q58FF8|H90B2_HUMAN	HSP90AB2I	2	2	1.70E+06	0.34
sp|P61626|LYSC_HUMAN	LYZ	2	2	1.00E+06	0.21
sp|Q07021|C1QBP_HUMAN	C1QBP	2	2	1.00E+06	0.21
sp|Q14240|IF4A2_HUMAN	EIF4A2	2	2	6.60E+05	0.14
sp|P19338|NUCL_HUMAN	NCL	2	2	2.40E+05	0.05
sp|P04040|CATA_HUMAN	CAT	2	2	4.20E+05	0.09
sp|P19474|RO52_HUMAN	TRIM21	2	2	7.40E+05	0.15
sp|Q01469|FABP5_HUMAN	FABP5	2	2	2.50E+06	0.51
sp|Q13867|BLMH_HUMAN	BLMH	2	2	7.90E+05	0.16
sp|Q15517|CDSN_HUMAN	CDSN	1	4	9.90E+06	2.05
sp|P05386|RLA1_HUMAN	RPLP1	1	3	3.80E+05	0.08
sp|Q9BVA1|TBB2B_HUMAN	TUBB2B	1	2	1.50E+06	0.30
sp|P62857|RS28_HUMAN	RPS28	1	2	4.30E+05	0.09
sp|Q13765|NACA_HUMAN	NACA	1	2	3.60E+05	0.08
sp|P30041|PRDX6_HUMAN	PRDX6	1	2	5.50E+05	0.11
sp|P01834|IGKC_HUMAN	IGKC	1	2	2.20E+06	0.45
sp|P51553|IDH3G_HUMAN	IDH3G	1	2	1.30E+07	2.77
sp|P23528|COF1_HUMAN	CFL1	1	1	1.50E+05	0.03
sp|P13929|ENOB_HUMAN	ENO3	1	1	3.70E+05	0.08
sp|P10599|THIO_HUMAN	TXN	1	1	1.30E+06	0.27
sp|P62424|RL7A_HUMAN	RPL7A	1	1	2.60E+05	0.05
sp|P14625|ENPL_HUMAN	HSP90B1	1	1	4.10E+05	0.08
sp|Q14568|HS902_HUMAN	HSP90AA2I	1	1	3.90E+05	0.08
sp|P46776|RL27A_HUMAN	RPL27A	1	1	3.60E+05	0.07
sp|P05387|RLA2_HUMAN	RPLP2	1	1	3.10E+05	0.06
sp|Q07020|RL18_HUMAN	RPL18	1	1	3.00E+05	0.06
sp|P50914|RL14_HUMAN	RPL14	1	1	1.90E+05	0.04
sp|P62913|RL11_HUMAN	RPL11	1	1	2.40E+05	0.05
sp|P28072|PSB6_HUMAN	PSMB6	1	1	2.10E+05	0.04
sp|P62753|RS6_HUMAN	RPS6	1	1	3.30E+05	0.07
sp|P22392|NDKB_HUMAN	NME2	1	1	2.70E+05	0.06
sp|Q9UI42|CBPA4_HUMAN	CPA4	1	1	2.60E+05	0.05
sp|P18124|RL7_HUMAN	RPL7	1	1	2.20E+05	0.05
sp|P62917|RL8_HUMAN	RPL8	1	1	2.00E+05	0.04
sp|P62701|RS4X_HUMAN	RPS4X	1	1	1.60E+05	0.03
sp|Q15431|SYCP1_HUMAN	SYCP1	1	1	3.80E+05	0.08
sp|Q96PP9|GBP4_HUMAN	GBP4	1	1	1.80E+05	0.04
sp|O43175|SERA_HUMAN	PHGDH	1	1	4.60E+05	0.09

Table S2. A list of all primers used in this study.

oligonucleotide name	sequence 5' to 3'
PGRMC1 forward	CTA CAA GGA CGA CGA TGA CAA GGG ATC CAT GGC TG
PGRMC1 reverse	CAC TAT AGA ATA GGG CCC TCT AGA TTA ATC
PGRMC2 reverse	CTA TAG AAT AGG GCC CTC TAG ATC AAT CCT GTT TAT TGT GAT
PGRMC1 1-170 reverse	GTA GTC TAG ATT ACA GTT TGC CCA CGT GAT G
PGRMC H1 forward	CAG CGG AAG GGG GAG GCT GGG AGA TTT TCA CGT CGC CGC TC
PGRMC H1 reverse	GAG CGG CGA CGT GAA AAT CTC CCA GCC TCC CCC TTC CGC TG
PGRMC H2 forward	GTG CTG CTG GGG GCC TAC CGG GGG GAC CAG CCG GCG GCC
PGRMC H2 reverse	GGC CGC CGG CTG GTC CCC CCG GTA GGC CCC CAG CAG CAC
PGRMC H3 forward	GCG GCG GGC TGC TGC ATG AGG CGG CGG CGG CGT TGG CGC TTC TG
PGRMC H3 reverse	CAG AAG CGC CAA CGC CGC CGC CGC CTC ATG CAG CAG CCCGCC GC
PGRMC 1-54 reverse	GAG GTC TAG ATT AGT CGT CGT CGC TGT CGC CGCT
PGRMC 21-195 forward	CGC TAG GAT CCC TGC TGC ATG AGA TTT TCA C
Xbp1 forward	GAA TGA AGT GAG GCC AGT GG
Xbp1 reverse	GGG GCT TGG TAT ATA TGT GG
