# Supplementary material for: PGRMC1 acts as a size-selective cargo receptor to drive ER-phagic clearance of mutant prohormones
Source: Nat Commun. 2021 Oct 13;12:5991. doi: 10.1038/s41467-021-26225-8 (PMC8514460; doi:10.1038/s41467-021-26225-8)
Supplement: Supplementary file 2 — Reporting Summary [file 41467_2021_26225_MOESM2_ESM.pdf]

nature research

Corresponding author(s): Billy Tsai

Last updated by author(s): 08/19/2021

Reporting Summary

Nature Research wishes to improve the reproducibility of the work that we publish. This form provides structure for consistency and transparency in reporting. For further information on Nature Research policies, see our Editorial Policies and the Editorial Policy Checklist.

Statistics

For all statistical analyses, confirm that the following items are present in the figure legend, table legend, main text, or Methods section.

n/a	Confirmed	
☐	☒	The exact sample size (n) for each experimental group/condition, given as a discrete number and unit of measurement
☐	☒	A statement on whether measurements were taken from distinct samples or whether the same sample was measured repeatedly
☐	☒	The statistical test(s) used AND whether they are one- or two-sided<br>*Only common tests should be described solely by name; describe more complex techniques in the Methods section.*
☒	☐	A description of all covariates tested
☒	☐	A description of any assumptions or corrections, such as tests of normality and adjustment for multiple comparisons
☐	☒	A full description of the statistical parameters including central tendency (e.g. means) or other basic estimates (e.g. regression coefficient) AND variation (e.g. standard deviation) or associated estimates of uncertainty (e.g. confidence intervals)
☐	☒	For null hypothesis testing, the test statistic (e.g. F, t, r) with confidence intervals, effect sizes, degrees of freedom and P value noted<br>*Give P values as exact values whenever suitable.*
☒	☐	For Bayesian analysis, information on the choice of priors and Markov chain Monte Carlo settings
☒	☐	For hierarchical and complex designs, identification of the appropriate level for tests and full reporting of outcomes
☒	☐	Estimates of effect sizes (e.g. Cohen's d, Pearson's r), indicating how they were calculated

Our web collection on statistics for biologists contains articles on many of the points above.

Software and code

Policy information about availability of computer code

Data collection	No software used for data collection
Data analysis	FIJI from NIH (version 1.52p), Genedoc (version 2.7)

For manuscripts utilizing custom algorithms or software that are central to the research but not yet described in published literature, software must be made available to editors and reviewers. We strongly encourage code deposition in a community repository (e.g. GitHub). See the Nature Research guidelines for submitting code & software for further information.

Data

Policy information about availability of data

All manuscripts must include a data availability statement. This statement should provide the following information, where applicable:

- Accession codes, unique identifiers, or web links for publicly available datasets
- A list of figures that have associated raw data
- A description of any restrictions on data availability

The mass spectrometry proteomics data have been deposited to the ProteomeXchange Consortium via the PRIDE 52 partner repository with the dataset identifier PXD024725 and 10.6019/PXD024725. All plasmids generated in this study have been made available on Addgene under the deposit #79811.

Field-specific reporting

Life sciences study design

All studies must disclose on these points even when the disclosure is negative.

Sample size	A sample size calculation was not performed. N=3 was used for each experiment in this work unless otherwise stated. N=3 allows for calculation of standard deviation use of the standard student's t-test.
Data exclusions	No data was excluded
Replication	All experiments were replicated at least three times and all attempts of replication were successful.
Randomization	Empty vector-expressing cells and scrambled siRNA-transfected cells were grouped as control. Cells expressing protein of interest (such as PGRMC1) and cells transfected with a siRNA against a protein of interest (such as RTN3 or PGRMC1) were grouped as test samples.
Blinding	Blinding was not performed as to guarantee that samples were properly processed. In order to prevent bias, raw data were analyzed by members of our laboratory community with the request to determine if the same conclusions could be made by an impartial scientist.

Reporting for specific materials, systems and methods

We require information from authors about some types of materials, experimental systems and methods used in many studies. Here, indicate whether each material, system or method listed is relevant to your study. If you are not sure if a list item applies to your research, read the appropriate section before selecting a response.

Materials & experimental systems

n/a	Involved in the study	
☐	☒	Antibodies
☐	☒	Eukaryotic cell lines
☒	☐	Palaeontology and archaeology
☐	☒	Animals and other organisms
☒	☐	Human research participants
☒	☐	Clinical data
☒	☐	Dual use research of concern

Methods

n/a	Involved in the study	
☒	☐	ChIP-seq
☒	☐	Flow cytometry
☒	☐	MRI-based neuroimaging

Antibodies

Antibodies used

primary antibodies:
1) anti-Myc; Immunology Consultants Laboratory, Cat #RMYC45A, 1:3000 dilution for western blot
2) anti-Myc; Santa Cruz Biotechnology; Cat# SC-40, 1:100 dilution for immunofluorescence
3) anti-FLAG; Millipore Sigma, Cat# F7425, RRID: AB_439687, 1:3000 dilution for western blot
4) anti-FLAG; Millipore Sigma, Cat# F3165, RRID: AB_259529, 1:3000 dilution for western blot
5) anti-HSP90; Santa Cruz Biotechnology, Cat# sc13119, RRID: AB_675659, 1:10000 dilution for western blot
6) anti-PGRMC1, polyclonal, generated in the laboratory of Dr. Peter Espenshade, 1:3000 dilution for western blot
7) anti-PGRMC1, Cell Signaling Technology, Cat #13856S, 1:3000 dilution for western blot, 1:100 dilution for immunofluorescence
8) anti-HA, Millipore Sigma, Cat# 11583816001, 1:3000 dilution for western blot
9) anti-RTN3, Bethyl Laboratories, Cat# A302--860A, 1:1000 dilution for western blot
10) anti-RTN3, Boster Biological Technology, Cat# PA2256; RRID: AB_2665372, 1:33 dilution for western blot
11) anti-Beclin1, MBL International, Cat# PD017, RRID:AB_1278767, 1:3000 dilution for western blot
12) anti-Hrd1, Proteintech Group, Cat# 13473--1--AP, RRID:AB_2287023, 1:3000 dilution for western blot
13) anti-Lamp1, Millipore Sigma, Cat #AB2971, 1:3000 dilution for western blot
14) anti-BiP, Proteintech Group, Cat# ab21685, RRID:AB_2119834, 1:10000 dilution for western blot
15) anti-CypB, Thermo Fisher Technology, Cat# PA1-027A, 1:1000 dilution for western blot
16) anti-proinsulin, Novus Biologicals, Cat# NB100-73013, 1:1000 dilution for western blot
17) anti-GFP, Proteintech Group, Cat# 66002--1--Ig, RRID:AB_11182611, 1:10000 dilution
18) anti-HA, Millipore Sigma, Cat#11867423001, 1:100 dilution for immunofluorescence

Secondary antibodies:
19) anti-mouse IgG peroxidase, Millipore Sigma Cat#A4416, 1:3000 dilution for western blot
20) anti-rabbit IgG peroxidase, Millipore Sigma, Cat#A4914, 1:3000 dilution for western blot
21) anti-mouse Alexa Fluor 594, Thermo Fisher, Cat#A-11032, 1:2000 dilution for immunofluorescence
22) anti-rat Alexa Fluor 488, Thermo Fisher, Cat#A-11006, 1:2000 dilution for immunofluorescence
23) anti-mouse Alexa Fluor 488, Thermo Fisher, Cat#A28175, 1:2000 dilution for immunofluorescence
24) anti-rabbit Alexa Fluor 488, Thermo Fisher, Cat #A11008, 1:2000 dilution for immunofluorescence

Validation	Primary antibodies:<br>1) anti-Myc; validation statement on the manufacturer website for use in immunofluorescence and western blot (epitope tag: species-independent)<br>2) anti-Myc; validation statement on the manufacturer website for use in immunofluoresence (epitope tag: species-independent)<br>3) anti-FLAG; validation statement on the manufacturer website for use in western blot and immunoprecipitation (epitope tag: species-independent)<br>4) anti-FLAG; validation statement on the manufacturer website for use in western blot and immunoprecipitation (epitope tag: species-independent)<br>5) anti-HSP90; validation statement on the manufacturer website for use in western blot in mouse and human cells.<br>6) anti-PGRMC1, validated by knockdown and knockout in this manuscript for western blot in human cells (Figure 2A, Figure 4E).<br>7) anti-PGRMC1, validation statement on the manufacturer website for western blot and immunofluoresence in human, rat, and muse cells.<br>8) anti-HA, validation statement on the manufacturer website for western blot and immunofluorescence (epitope tag: species-independent)<br>9) anti-RTN3, validation statement on the manufacturer website for IP and western blot in human cells<br>10) anti-RTN3, validation statement on the manufacturer website for immunofluoresence and western blot in human, mouse, and rat cells<br>11) anti-Beclin1,validation statement on the manufacturer website for western blot in human, mouse, and rat cells<br>12) anti-Hrd1, validation statement on the manufacturer website for western blot in human, mouse, and rat cells<br>13) anti-Lamp1, validation statement on the manufacturer website for western blot in human, mouse, and rat cells<br>14) anti-BiP, validation statement on the manufacturer website for western blot in human, rat, and mouse cells<br>15) anti-CypB, validation statement on the manufacturer website for western blot in human, mouse, and rat cells<br>16) anti-proinsulin, validation statement on the manufacturer website for western blot in human and rat cells<br>17) anti-GFP, validation statement on the manufacturer website for western blot (epitope tag: species-independent).<br>18) anti-HA, validation statement on the manufacturer website for western blot and immunofluorescence (epitope tag, species independent)

Eukaryotic cell lines

Policy information about cell lines

Cell line source(s)	1) HEK 293T (ATCC)<br>2) COS-7 (ATCC)<br>3) INS1E (Laboratory of Claes B Wolheim, PMID: 1370150)<br>4) INS 832/13 (Laboratory of Christopher Newgard, PMID: 10868964)
Authentication	Cells are authenticated by morphology, karyotyping and PCR based approaches according to ATCC.
Mycoplasma contamination	All cells are negative from Mycoplasma contamination.
Commonly misidentified lines<br>(See ICLAC register)	No commonly misidentified cell lines were used.

Animals and other organisms

Policy information about studies involving animals; ARRIVE guidelines recommended for reporting animal research

Laboratory animals	Male and female B6 mice aged 6 weeks were used. Mice were housed at 72 degrees F, 30-70% humidity, light cycle of 7am-7pm.
Wild animals	No wild animals were used in this study.
Field-collected samples	No field collected samples were used in this study.
Ethics oversight	Institutional Animal Care and Use Committee (IACUC) has approved this study protocol for use of animals to meet the state and federal ethical standards.

Note that full information on the approval of the study protocol must also be provided in the manuscript.
